# Natural compounds for non-small cell lung cancer treatment: focus on the EGFR signaling pathway

**DOI:** 10.3389/fphar.2026.1758414

**Published:** 2026-03-27

**Authors:** Baibai Ye, Qi Xiao

**Affiliations:** 1 The Sixth People's Hospital of Huizhou, Huizhou, Guangdong, China; 2 Hunan University of Chinese Medicine, Changsha, Hunan, China

**Keywords:** drug resistance, EGFR signaling pathway, natural compounds, NSCLC, precision therapy

## Abstract

The EGFR signaling pathway is a critical driver in the occurrence and development of non-small cell lung cancer (NSCLC). However, the inevitable development of acquired resistance to EGFR tyrosine kinase inhibitor (TKI) poses a major therapeutic challenge. Natural compounds, with their intrinsic multi-target capabilities and favorable safety profiles, represent a promising strategy for overcoming this resistance. This review provides a critical synthesis of current evidence for over 33 representative natural compounds—spanning alkaloids, terpenoids, flavonoids, and polyphenols—with a focus on their mechanisms for enhancing TKI efficacy. These include direct inhibition of EGFR activation, regulation of key downstream signaling pathways, and induction of programmed cell death. Furthermore, it also examine how emerging approaches such as nano-delivery systems can overcome the pharmacokinetic limitations of these compounds. Ultimately, this review provides a novel, strategy-oriented perspective by framing natural compounds not merely as standalone agents, but as essential components of rational combination therapies, thereby offering a fresh roadmap for their clinical translation in precision oncology for NSCLC.

## 1 Introduction

Lung cancer remains the leading cause of cancer-related mortality worldwide, with non-small cell lung cancer (NSCLC) accounting for approximately 85% of cases (Jeon et al., 2025; Huang et al., 2025). The discovery of activating mutations in the epidermal growth factor receptor (EGFR) gene, such as exon 19 deletions and the L858R point mutation, has transformed the therapeutic landscape. EGFR tyrosine kinase inhibitors (TKIs), including gefitinib, erlotinib, and osimertinib, have become the standard first-line therapy for patients with EGFR-mutant NSCLC, demonstrating remarkable clinical efficacy (Zhou et al., 2025).

However, the long-term benefit of EGFR-TKIs is invariably limited by the development of acquired resistance, which now represents a paramount clinical challenge (Riely et al., 2024; Bouchard and Daaboul, 2025). Resistance mechanisms are complex and heterogeneous, encompassing on-target secondary EGFR mutations (e.g., T790M, C797S), activation of bypass signaling pathways (e.g., MET, AXL), and phenotypic transformations such as epithelial-mesenchymal transition (EMT). This complexity underscores the limitations of sequential single-target therapies and creates an urgent need for innovative strategies that can simultaneously address multiple facets of the resistance network.

In this context, plant-derived natural compounds emerge as a compelling source of novel therapeutic agents. Their diverse chemical structures, intrinsic multi-target capabilities, and generally favorable safety profiles position them as ideal candidates for developing rational combination therapies aimed at overcoming TKIs resistance (Wang et al., 2016; Andrés et al., 2024; Imtiaz et al., 2025). Rather than acting as mere substitutes for existing TKIs, their strategic value may lie in their ability to modulate the core EGFR pathway alongside its crosstalk networks, thereby resensitizing resistant tumors.

To systematically evaluate this potential, a structured literature search was conducted following a predefined strategy. Databases including PubMed, Google Scholar, and Web of Science were queried for publications from January 2010 to June 2025, using a search strategy that combined key terms from three conceptual groups: (1) disease context (“non-small cell lung cancer” OR “NSCLC”), (2) molecular target/pathology (“EGFR” OR “epidermal growth factor receptor” OR “tyrosine kinase inhibitor resistance”), and (3) intervention (“natural compound” OR “phytochemical” OR “alkaloid” OR “terpenoid” OR “flavonoid” OR “polyphenol”). Studies were screened according to predefined inclusion criteria—original research or seminal reviews focusing on defined natural compounds and their effects on EGFR signaling or TKI resistance in NSCLC—and exclusion criteria, such as non-English publications, studies on other cancer types without direct NSCLC relevance, and reports on uncharacterized herbal mixtures. Eligible studies were critically appraised based on model systems, experimental robustness, mechanistic clarity, and translational relevance, thereby informing a synthesis that highlights both the promise and limitations of the current evidence.

This review, therefore, is framed around a central strategic perspective: to critically assess the evidence for natural compounds—spanning alkaloids, terpenoids, flavonoids, and polyphenols—as multi-targeted partners in combination regimens designed to prevent or reverse EGFR TKI resistance. By synthesizing their mechanisms of action and translational challenges, we aim to provide a roadmap for integrating these promising agents into the precision oncology paradigm for NSCLC.

## 2 EGFR signaling pathway and its role in NSCLC resistance

The EGFR pathway is a central regulator of cellular growth and survival, and its dysregulation is a principal oncogenic driver in NSCLC. Therapeutic targeting of mutant EGFR with TKIs initially yields high response rates, but the inevitable emergence of acquired resistance remains a major clinical challenge. This section provides a focused overview of EGFR signaling, highlighting the structural and regulatory features most relevant to NSCLC pathogenesis and the development of TKI resistance.

### 2.1 Key features of EGFR structure and oncogenic mutations

**FIGURE 1:**
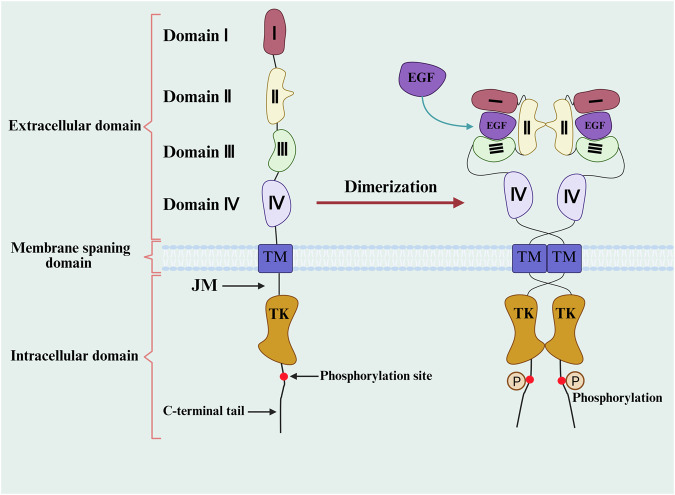
The structure of EGFR and activated EGFR.

EGFR is a transmembrane receptor with an extracellular ligand-binding domain, a transmembrane anchor, and an intracellular tyrosine kinase domain (Figure 1). Oncogenesis in NSCLC is frequently driven by activating mutations within the kinase domain, most commonly exon 19 deletions and the L858R point mutation, which confer constitutive signaling and sensitivity to first- and second-generation TKIs (Bellevicine et al., 2014; Castellanos et al., 2017; Rossi and Galetta, 2022). The acquisition of secondary mutations, such as T790M, restores ATP affinity and is a classic mechanism of resistance, later targeted by third-generation TKIs like osimertinib.

### 2.2 Core signaling cascades and dysregulation in resistance

**FIGURE 2:**
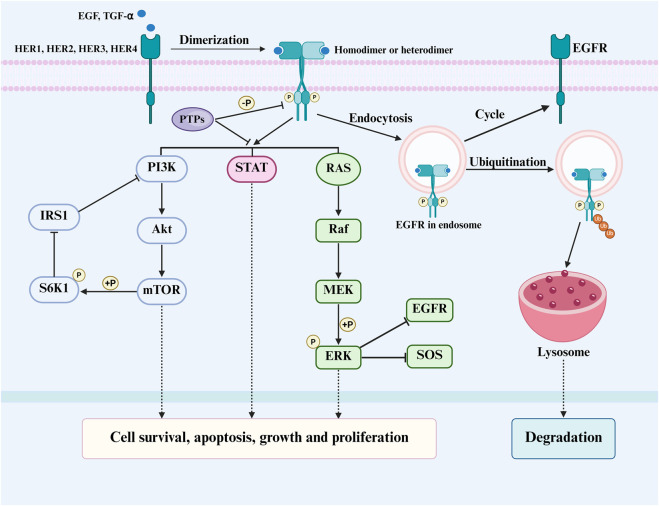
The composition, activation, and negative regulation of the EGFR signaling pathway.

Ligand binding induces EGFR dimerization and autophosphorylation, recruiting adaptor proteins to activate downstream pathways critical for tumor survival: the RAS/RAF/MEK/ERK (MAPK) and PI3K/Akt/mTOR pathways (Wang et al., 2020; Murphrey et al., 2025) (Figure 2). In the context of resistance, these cascades can be reactivated despite EGFR inhibition. For instance, mutated EGFR variants often exhibit impaired endocytosis, leading to prolonged receptor retention at the membrane and sustained downstream signal output (Sorkin and Duex, 2010; Wee and Wang, 2017). Moreover, persistent activation of pathways like JAK/STAT can promote the expression of anti-apoptotic genes, further contributing to treatment evasion.

### 2.3 Mechanisms of signal attenuation and their failure

Under physiological conditions, EGFR signaling is precisely controlled through a multi-layered attenuation system to prevent sustained proliferative signaling (Schultz et al., 2023). The primary mechanism involves receptor downregulation via clathrin-mediated endocytosis, where activated EGFR is internalized and sorted for either recycling to the membrane or degradation in lysosomes following ubiquitination by E3 ligases like Cbl (Salih et al., 2025). This degradation pathway, mediated by the ESCRT complex, can shorten EGFR’s half-life to under 1 h, providing a rapid termination mechanism for mitogenic signals. Concurrently, protein tyrosine phosphatases (PTPs) function as critical negative regulators by directly dephosphorylating activated EGFR and its downstream effectors, thereby resetting the signaling cascade (Figure 2). Furthermore, intrinsic negative feedback loops-such as ERK-mediated inhibition of upstream SOS and EGFR, or mTORC1/S6K1-induced suppression of IRS1-create self-limiting circuits that maintain signaling homeostasis.

In NSCLC, particularly in the context of TKI resistance, these regulatory mechanisms are frequently subverted. Mutant EGFR variants often exhibit impaired endocytic trafficking and defective ubiquitination, leading to prolonged membrane retention and constitutive signaling (Xia et al., 2025). The tumor microenvironment (TME) may further suppress PTP activity or expression, disrupting the phosphorylation-dephosphorylation balance. Additionally, chronic TKI exposure can dysregulate feedback mechanisms, enabling adaptive survival signaling through alternative nodes. This collective failure of endogenous attenuation not only contributes to oncogenic progression but also establishes a permissive landscape for resistance, wherein tumor cells evade pharmacological inhibition through reinforced signaling networks. Understanding these compromised regulatory checkpoints is therefore essential for designing strategies to restore pathway control.

### 2.4 Crosstalk with bypass pathways: the nexus of resistance

A primary cause of TKI failure is the activation of alternative signaling networks that bypass EGFR dependence. The MET pathway, through amplification or overexpression, reactivates the shared PI3K/Akt and MAPK cascades, sustaining survival signals independently of EGFR inhibition (Breindel et al., 2013; Reznik et al., 2008). Simultaneously, dynamic crosstalk with the TME establishes a resilient pro-tumorigenic loop: EGFR signaling upregulates immune checkpoint molecules such as PD-L1 to facilitate immune evasion, while inflammatory cytokines from the TME reciprocally enhance EGFR pathway activity, further entrenching therapeutic resistance (Gu et al., 2025). Furthermore, under TKI-induced stress, tumor cells may activate protective autophagy as an adaptive survival mechanism, revealing another actionable axis for therapeutic intervention (Zaryouh et al., 2022). Collectively, this network of bypass signaling, microenvironmental interplay, and stress adaptation constitutes a multifaceted barrier to durable EGFR inhibition. It thus provides a compelling biological rationale for pursuing multi-target therapeutic strategies—including natural compounds with polypharmacological profiles—capable of concurrently suppressing EGFR along with these key resistance axes (Jänne et al., 2015; Soria et al., 2018).

## 3 Therapeutic challenges and strategic opportunities: rationale for novel interventions

EGFR-TKIs represent a landmark achievement in the precision therapy of EGFR-mutant NSCLC. However, as delineated in Section 2, the long-term efficacy of these agents is universally compromised by the emergence of acquired resistance. This resistance is not monolithic but manifests as a complex network encompassing on-target secondary mutations, activation of bypass tracks, phenotypic transformation, and adaptive remodeling of the tumor microenvironment. This multifaceted nature means that sequential targeting of single resistance mechanisms often leads only to the emergence of the next.

Consequently, next-generation synthetic TKIs and combination regimens, while advancing the field, frequently encounter inherent limitations. These include a narrow spectrum of activity against specific resistance mechanisms, the potential to inadvertently select for novel resistant clones, and the compounded systemic toxicities associated with targeted drug combinations. There exists, therefore, a clear and pressing therapeutic gap: the need for agents capable of concurrently modulating multiple nodes within the oncogenic signaling network to preempt or overcome polyclonal resistance, all while maintaining a manageable safety profile.

This gap presents a strategic opportunity for natural compounds. Inherently equipped with polypharmacological profiles, these plant-derived molecules can simultaneously influence multiple pathways—such as the core EGFR axis, its key downstream effectors, and critical bypass signals. Coupled with their historically favorable toxicity profiles, natural compounds offer a compelling rationale as backbone agents or sensitizers in next-generation combination strategies aimed at durable resistance control. Therefore, rather than presenting a mere catalog of compounds, the following sections are organized to critically evaluate each major class (alkaloids, terpenoids, flavonoids, and polyphenols) through a resistance-centric lens. We will focus on their documented abilities to: (1) co-target core EGFR signaling and key resistance-associated bypass pathways; and (2) synergize with existing TKIs to restore therapeutic sensitivity. This analytical framework directly addresses the multifaceted resistance network outlined above, providing a strategic assessment of their translational potential.

## 4 Natural compounds targeting the EGFR signaling pathway in NSCLC therapy

The inherent polypharmacology of natural compounds offers a unique strategic advantage against the multifaceted network of EGFR TKI resistance. Unlike single-target synthetic drugs, these molecules can simultaneously modulate the core EGFR pathway and its key bypass tracks, positioning them as ideal candidates for rational combination therapies. This section moves beyond a mere cataloging of effects to provide a critical synthesis of the major classes of natural compounds—alkaloids, terpenoids, flavonoids, and polyphenols-evaluating their mechanisms, preclinical evidence, and most importantly, the translational challenges that must be addressed to realize their clinical potential in NSCLC. A comparative overview of representative compounds, their primary resistance targets, and evidence levels is provided in Table 1.

**TABLE 1:** Natural compounds targeting the EGFR signaling pathway in NSCLC therapy.

No.	Compound name	Formula	Classification	Evidence level	Upregulates targets	Downregulates targets	Main effects	Effects on EGFR TKI resistance	References
1	Berberine	C_20_H_18_NO_4_^+^	Alkaloid	*In vitro* and *in vivo*	EMT.	p-EGFR, p-Akt and p-mTOR.	Inhibits proliferation, migration, invasion; induces autophagy or apoptosis	Synergistic sensitization; reversal resistance caused by MET amplification	Chen et al. (2021), Zhang et al. (2025), Ming et al. (2024)
2	Piperlongumine	C_17_H_19_NO_5_	Alkaloid	*In vitro* and *in vivo*	Mcl-1 ubiquitination	EGFR mutants, EGFR and PI3K/Akt	Induces apoptosis	Synergistic sensitization; overcoming resistance caused by T790M mutations	Liu et al. (2025), Wang S. et al. (2025)
3	Matrine	C_15_H_24_N_2_O	Alkaloid	*In vitro* and *in vivo*	—	IL-6,JAK1 and STAT3	Induces apoptosis	Synergistic sensitization	Chen et al. (2017)
4	Oxymatrine	C_15_H_24_N_2_O_2_	Alkaloid	*In vitro* and *in vivo*	—	p-EGFR, p-Akt and p-ERK.	Induces cell cycle arrest	Synergistic sensitization	Li W. et al. (2018)
5	Sanguinarine	C_20_H_14_NO_4_^+^	Alkaloid	*In vitro* and *in* *vivo*	NOX3	MsrA	Promote oxidation and degradation of EGFR T790M mutant	Overcomes TKIs resistance, notably by reversing the T790M-mediated mechanism	Leung et al. (2016), Saini et al. (2022)
6	Griffithazanone A	C_14_H_11_NO_4_	Alkaloid	*In vitro* and *in vivo*	—	PIM1	Induces apoptosis	Synergistic sensitization; reversal of Osimertinib resistance	Xiao et al. (2024)
7	Celastrol	C_29_H_38_O_4_	Triterpenoid	*In vitro* and *in vivo*	—	EGFR, PI3K/Akt and RAS/MAPK.	Induces EGFR degradation; inhibits proliferation; induces apoptosis	Synergistic sensitization; overcomes TKIs resistance	Xu et al. (2016), Dai et al. (2021), Wang et al. (2018), Lee et al. (2019)
8	Ginsenoside Rg3	C_42_H_72_O_13_	Triterpenoid	*In vitro*, *in vivo* and clinical	—	PD-L1 glycosylation and HIF-1α/VEGF/EGFR.	Induces autophagy; inhibits proliferation	Synergistic sensitization; overcomes osimertinib resistance	Wang X. J. et al. (2019), Liang et al. (2021), Wang et al. (2024a)
9	β-elemene	C_15_H_24_	Triterpenoid	*In vitro* and *in vivo*	AMPK/MAPK.	EZH2, IncRNA H19 and TFEB.	Inhibits proliferation; induces apoptosis; induces ferroptosis and autophagy	Synergistic sensitization; reversal of TKIs resistance	Wang et al. (2021), Xu et al. (2023), Zhang R.et al. (2024), Zhao et al. (2024)
10	Ursolic acid	C_30_H_48_O_3_	Triterpenoid	*In vitro*, *in vivo* and clinical	—	β-catenin/TCF4/CT45A2, EGFR and JAK2/STAT3	Induces apoptosis; inhibits proliferation, migration and invasion	Synergistic sensitization; overcomes TKIs resistance	Yang et al. (2019), Kang et al. (2021)
11	Betulinic acid	C_30_H_48_O_3_	Triterpenoid	*In vitro*	—	EGFR and PI3K/Akt/mTOR.	Inhibits proliferation; induces autophagy and apoptosis; induces cell cycle arrest	Synergistic sensitization; overcomes TKIs primary resistance	Ko et al. (2018), Torres-Martinez et al. (2023), Wang H. et al. (2024)
12	Oleanolic acid	C_30_H_48_O_3_	Triterpenoid	*In vitro*	—	PI3K/Akt/mTOR.	Induces apoptosis	—	Zhou et al. (2024), Chen et al. (2019a)
13	Oridonin	C_20_H_28_O_6_	Triterpenoid	*In vitro* and *in* *vivo*	—	EGFR/ERK/MMP-12 and CIP2A/PP2A/Akt	Inhibits proliferation	Overcomes Gefitinib resistance	Xiao et al. (2016)
14	Cucurbitacin B	C_32_H_46_O_8_	Triterpenoid	*In vitro* and *in vivo*	—	p-EGFR, PI3K/mTOR and STAT3	Inhibits proliferation; induces apoptosis	—	Khan et al. (2017), Liu et al. (2019)
15	Dihydroartemisinin	C_15_H_24_O_5_	Triterpenoid	*In vitro* and *in vivo*	ROS degradation	—	Induces ferroptosis	Overcomes EGFR TKI resistance	Cai et al. (2021), Lai et al. (2023)
16	Costunolide	C_15_H_20_O_2_	Triterpenoid	*In vitro*	—	MEK1 and AKT1/2	—	Overcomes osimertinib resistance	Tian et al. (2022)
17	Quercetin	C_15_H_10_O_7_	Flavonoid	*In vitro* and *in vivo*	—	EGFR, ERK/MEK, Akt/S6K NF-κB and multi-targets RTKs (c-Met, Her-2, AXL, IGF1R)	Inhibits proliferation; induces apoptosis and autophagy	Synergistic sensitization; reversal of TKIs resistance	Ge et al. (2023), Chan et al. (2023), Jiao et al. (2023), Alam et al. (2022), Ramirez et al. (2021)
18	Hyperoside	C_21_H_20_O_12_	Flavnoid	*In vitro* and *in vivo*	FoxO1	IncRNA CCAT1	Inhibits proliferation; induces apoptosis	—	Hu Z. et al. (2020)
19	Luteolin	C_15_H_10_O_6_	Flavnoid	*In vitro* and *in vivo*	—	EGFR, ERK, Akt/mTOR and NF-κB	Induces apoptosis and autophagy	Synergistic sensitization; overcomes TKIs resistance	Zhang J. et al. (2024), Zhang et al. (2022), Ambrose et al. (2018), Hong et al. (2014), Huang G. et al. (2023), Ganthala et al. (2022)
20	Apigenin	C_15_H_10_O_5_	Flavonoid	*In vitro* and *in vivo*	—	p-EGFR, EGFR, HIF-1α, c-Myc, PI3K/Akt and RAS/RAF/MEK/ERK.	Inhibits proliferation, migration, invasion; induces apoptosis	Synergistic sensitization; overcomes resistance caused by L858R/T790M mutations	Chang et al. (2018), Chen et al. (2019b)
21	Dihydromyricetin	C_15_H_12_O_8_	Flavonoid	*In vitro* and *in vivo*	​	EGFR, Akt, survivin	Induces apoptosis	Overcomes TKIs resistance	Li et al. (2023)
22	Hydroxygenkwanin	C_16_H_12_O_6_	Flavonoid	*In vitro* and *in vivo*	EGFR ubiquitination and degradation	STAT3, Akt and ERK.	Induces apoptosis	Overcomes TKIs resistance	Leu et al. (2020)
23	Curcumin	C_21_H_20_O_6_	Polyphenol	*In vitro* and *in vivo*	—	EGFR, ERK/MEK, Akt/S6K and multi-targets RTKs (c-Met, Her-2, AXL, IGF1R)	Inhibits proliferation; induces apoptosis and autophagy	Synergistic sensitization; reversal of TKIs resistance	Ye et al. (2012), Chen P. et al. (2019), Zhang et al. (2019), Li et al. (2013), Wada et al. (2015)
24	Resveratrol	C_14_H_12_O_3_	Polyphenol	*In vitro*	AMPK.	p-EGFR, EGFR, ERK/MEK, PI3K/Akt and Akt/mTOR.	Inhibits proliferation; induces apoptosis, autophagy and senescence	Synergistic sensitization; reversal of TKIs resistance	Li W. et al. (2016), Zhu et al. (2015), Lu et al. (2019), Zhao et al. (2023), Fan et al. (2015)
25	EGCG	C_22_H_18_O_11_	Polyphenol	*In vitro* and *in vivo*	EGFR degradation	MAPK, PI3K/Akt and STAT3	Inhibits proliferation, migration; induces cell cycle arrest and apoptosis	Synergistic sensitization; reversal of TKIs resistance	Minnelli et al. (2020), Meng et al. (2019), Minnelli et al. (2021), Polonio-Alcalá et al. (2025)
26	Gallic Acid	C_7_H_6_O_5_	Polyphenol	*In vitro* and *in vivo*	EGFR degradation	p-EGFR, PI3K/Akt and RAS/ERK.	Inhibits proliferation; induces apoptosis	Overcomes TKIs resistance	Kang et al. (2020), Wang and Bao (2020), Phan et al. (2016)
27	Ellagic Acid	C_14_H_6_O_8_	Polyphenol	*In vitro* and *in vivo*	—	EGFR.	Inhibits proliferation; induces apoptosis	Synergistic sensitization; reversal of TKIs resistance	Ayaz et al. (2022), Xie et al. (2020b)
28	Shikonin	C_16_H_16_O_5_	Others	*In vitro* and *in vivo*	ROS and EGFR degradation	PI3K/Akt and MEK/ERK	Induces apoptosis	Synergistic sensitization	Li et al. (2017), Hsieh et al. (2017), Hu et al. (2020b)
29	Silibinin	C_25_H_22_O_10_	Others	*In vitro* and *in vivo*	—	EGFR, EMT, PI3K/Akt and JAK/STAT.	Inhibits proliferation, migration and invasion	Synergistic sensitization; overcomes acquired resistance	Wang (2025), Rugamba et al. (2021)
30	Psorachromene	C_20_H_18_O_4_	Others	*In vitro*	—	EGFR.	Inhibits proliferation	—	Wang et al. (2022)
31	Xanthohumol	C_21_H_22_O_5_	Others	*In vitro* and *in vivo*	Ets-1 ubiquitination	c-Met.	Inhibits proliferation	Overcomes Osimertinib resistance	Ma et al. (2024)
32	Genipin	C_11_H_14_O_5_	Others	*In vitro*	—	EGFR/JAK1/STAT3	Inhibits migration and invasion; induces apoptosis	—	Kim et al. (2025)
33	α-Mangostin	C_24_H_26_O_6_	Others	*In vitro*	—	EGFR/STAT3	Inhibits proliferation	—	Wang J. et al. (2025)

### 4.1 Alkaloids

Berberine (BBR) has demonstrated the ability to inhibit the EGFR pathway and synergize with EGFR-TKIs in preclinical NSCLC models (Cuan et al., 2023; Chen et al., 2021; Chen et al., 2022). However, it is crucial to note that these promising results are largely derived from *in vitro* and animal studies. A significant translational challenge for BBR is its poor oral bioavailability and rapid systemic elimination, which may limit its effective concentration at the tumor site. While some studies report low cytotoxicity in certain normal cells (Zhang et al., 2025), a systematic and comprehensive toxicity profile, including potential hepatotoxicity or gastrointestinal effects at therapeutic doses, has not been fully established in the context of NSCLC treatment. Furthermore, its potential interactions with co-administered drugs via cytochrome P450 enzymes warrant investigation. Therefore, despite its promising multi-target mechanism, BBR’s clinical application in NSCLC is contingent upon overcoming these pharmacokinetic hurdles and obtaining robust safety data from well-designed clinical trials.

Piperlongumine (PL) has emerged as a candidate with a dual mechanism against EGFR TKI resistance, functioning as a direct inhibitor of both wild-type and mutant EGFR while concurrently promoting the degradation of the anti-apoptotic protein Mcl-1 via the Akt/GSK3β axis to induce mitochondrial apoptosis (Liu et al., 2025). Preclinical studies indicate that PL synergizes with gefitinib or erlotinib and demonstrates antitumor activity in osimertinib-resistant xenograft models (Wang S. et al., 2025; Modi and Andey, 2024). However, this promising preclinical profile must be critically evaluated: its efficacy in animal models does not guarantee human translational success, as critical pharmacokinetic parameters-including bioavailability, tissue distribution, and human-specific metabolism-remain largely uncharacterized. Furthermore, claims of favorable tolerability are based on limited short-term animal observations and do not constitute a comprehensive safety assessment, leaving potential organ toxicity and off-target effects unexamined. Thus, while PL represents a mechanistically compelling preclinical lead, its advancement as a viable clinical strategy necessitates rigorous pharmacologic and toxicologic investigation to address these fundamental translational hurdles.

Matrine, oxymatrine, sanguinarine, and griffithazanone A exemplify the diverse mechanisms through which alkaloids may counteract EGFR TKI resistance. Matrine appears to modulate the tumor microenvironment and apoptosis in T790M-mutant cells by reducing IL-6 and downregulating Bcl-2 via the JAK1/STAT3 pathway, demonstrating preclinical synergy with afatinib (Chen et al., 2017). Oxymatrine directly suppresses phosphorylation of diverse EGFR forms (wild-type, L858R/T790M, Ex19del) and their downstream Akt/ERK activity, leading to cell cycle arrest (Li W. et al., 2018). Sanguinarine employs a redox-based strategy, inducing ROS to specifically degrade the EGFR T790M mutant (Leung et al., 2016), and computational studies suggest high-affinity binding to EGFR (Saini et al., 2022). Griffithazanone A, although not a direct EGFR inhibitor, targets the upstream kinase PIM1 to enhance apoptosis and shows synergistic effects with gefitinib and osimertinib (Xiao et al., 2024). However, the translational potential of these compounds is constrained by significant gaps in characterization. For matrine and oxymatrine, promising *in vivo* efficacy and reported low toxicity in xenograft models remain preliminary, as critical pharmacokinetic parameters—including oral bioavailability and human metabolic profiles—are undefined, and their long-term safety is unestablished. Sanguinarine’s ROS-mediated mechanism raises inherent safety concerns regarding potential off-target oxidative damage to normal tissues, a risk not yet rigorously evaluated. Its computationally predicted high binding affinity also requires empirical validation in relevant biological systems. Similarly, griffithazanone A’s action through PIM1 inhibition may lead to broader signaling perturbations with unknown systemic consequences. Collectively, while these alkaloids present mechanistically compelling preclinical leads, their progression is hindered by a lack of comprehensive drug-like property assessment and safety pharmacology, underscoring the necessity of addressing these fundamental gaps prior to clinical development.

### 4.2 Terpenoids

Celastrol, a natural triterpene, demonstrates a multi-mechanistic approach to overcoming EGFR TKI resistance in preclinical NSCLC models by inducing EGFR degradation to suppress downstream PI3K/Akt and RAS/MAPK signaling, thereby inhibiting proliferation and promoting apoptosis (Xu et al., 2016; Dai et al., 2021). It exhibits synergistic effects with gefitinib or osimertinib, enhancing tumor growth suppression *in vivo* (Wang et al., 2018; Lee et al., 2019; Fan et al., 2014), and operates through additional pathways including autophagy regulation and downregulation of the resistance-linked protein SHOC2 (Xu et al., 2016; Terai et al., 2021). Advanced delivery strategies, such as glutathione-responsive nano-drug systems co-delivering celastrol and gefitinib, have been developed to concurrently target Hsp90 and degrade client proteins like EGFR and Akt, showing promise against resistant cells in preclinical settings (Xie X. et al., 2020). However, the translation of celastrol faces a critical barrier rooted in its well-documented narrow therapeutic index and associated systemic toxicity concerns, including potential hepatotoxicity and cardiotoxicity. While innovative nano-formulations aim to mitigate these risks by improving tumor targeting, they remain at an early experimental stage. Consequently, celastrol exemplifies a potent natural lead compound whose clinical advancement is contingent not merely on its multi-target efficacy but on the successful development of delivery platforms that can decisively separate its antitumor activity from its adverse safety profile in humans.

Ginsenoside Rg3 represents a notable case where a natural compound has generated both preclinical mechanistic insights and preliminary clinical observations in NSCLC. A clinical retrospective study reported that combining EGFR-TKIs with Rg3 significantly prolonged progression-free survival (PFS) and improved objective response rates (ORR), suggesting a potential sensitizing effect in patients (Li Y. et al., 2016). Mechanistically, preclinical research indicates Rg3 operates through multiple avenues: it enhances TKI sensitivity by inhibiting protective autophagy, directly suppresses EGFR-mediated proliferation signals, modulates the tumor immune microenvironment by inhibiting PD-L1 glycosylation, and when formulated as a nano-drug with osimertinib, targets the HIF-1α/VEGF/EGFR axis to overcome resistance (Wang X. J. et al., 2019; Liang et al., 2021; Wang W. et al., 2024; Jiang et al., 2025). Furthermore, Rg3 has been shown to reduce EGFR gene copy number and protein expression in mutant cells (Lv et al., 2025). While the clinical data provides a valuable signal, it originates from a retrospective analysis and requires prospective validation. The major impediment to Rg3’s translation is its exceptionally poor oral bioavailability, which severely limits the clinical relevance of its preclinical activity. Although innovative nano-formulations represent a promising solution to this pharmacokinetic challenge, they are not yet clinically established (Wang W. et al., 2024). Therefore, the future of Rg3 depends on successfully addressing its delivery limitations through pharmaceutical engineering and confirming its synergistic benefits in controlled clinical trials.

β-Elemene demonstrates a multi-pathway approach to overcoming EGFR TKI resistance in preclinical NSCLC models. Research indicates its activity is mediated through the downregulation of EZH2 to enhance gefitinib’s effects, activation of AMPK and MAPK pathways to promote apoptosis in resistant cells, and modulation of lncRNA H19 to induce ferroptosis and suppress protective autophagy, thereby increasing erlotinib sensitivity (Cheng et al., 2018; Wang et al., 2021; Xu et al., 2023; Zhang R. et al., 2024). A distinct mechanism involving TFEB-mediated GPX4 degradation has also been identified, suggesting potential utility in EGFR-wildtype contexts (Zhao et al., 2024). Despite promising *in vitro* synergy, β-Elemene’s translation faces defined challenges. Its established clinical use as an intravenous emulsion for palliative care underscores its poor pharmacokinetic properties, including negligible oral bioavailability. The relevance of its diverse cellular mechanisms to human tumors, and its safety profile in combination with targeted therapies, remain unvalidated. Thus, while mechanistically interesting, β-Elemene requires advanced formulation strategies and rigorous clinical evaluation to assess its utility in modern TKI-based regimens.

Ursolic acid (UA) demonstrates multi-target activity against EGFR TKI resistant NSCLC in preclinical studies. It shows specificity against T790M-mutant cells by downregulating the β-catenin/TCF4/CT45A2 pathway to induce apoptosis (Yang et al., 2019), directly binds to EGFR to inhibit its phosphorylation and downstream JAK2/STAT3 signaling, concurrently reducing PD-L1 expression, and has been engineered into nano-formulations for combination with chemotherapeutics or erlotinib to enhance delivery and efficacy (Yang et al., 2019; Kang et al., 2021; Fu et al., 2022; Wang Z. et al., 2025). The multi-mechanistic profile of UA is pharmacologically promising, yet its development is constrained by inherent physicochemical and pharmacokinetic limitations. As a typical pentacyclic triterpenoid, UA suffers from extremely low aqueous solubility and poor oral bioavailability, which fundamentally restrict its effective *in vivo* application. The reported nano-formulations represent essential but early-stage solutions to this delivery problem (Fu et al., 2022; Wang Z. et al., 2025). Furthermore, while UA appears to modulate immune checkpoints like PD-L1, the functional consequences and potential immunomodulatory risks of this effect in a therapeutic context are unexplored (Kang et al., 2021). Therefore, UA exemplifies a compound whose translational path is unequivocally dependent on pharmaceutical innovation to overcome delivery barriers, and whose promising multi-target pharmacology requires validation in models that account for its formulated pharmacokinetics.

Betulinic acid (BA) exhibits promising anti-NSCLC activity through EGFR pathway modulation. Preclinical studies indicate BA enhances apoptosis and autophagy in TKI-resistant cells when combined with EGFR-TKIs (Ko et al., 2018). A BSA-based nanocarrier co-delivering BA and doxorubicin showed synergistic effects and EGFR downregulation (Torres-Martinez et al., 2023). Notably, a 2024 study combining molecular docking and experimental validation demonstrated that BA directly binds to the ATP-binding site of wild-type EGFR, inhibiting its autophosphorylation and downstream PI3K-Akt-mTOR signaling, thereby synergizing with TKIs to overcome primary resistance in wt-EGFR models (Wang H. et al., 2024). While BA’s mechanism of action—particularly its direct binding to wt-EGFR—is mechanistically compelling, its clinical translation faces defined pharmacokinetic hurdles. Like many triterpenoids, BA is expected to have poor aqueous solubility and suboptimal bioavailability, which are not adequately addressed in the current literature. The demonstrated nano-formulation is a necessary but preliminary step (Torres-Martinez et al., 2023). Furthermore, the specificity of its binding to wt-EGFR over mutant forms, and the potential for off-target effects given its multi-pathway activity, require careful evaluation. Therefore, BA represents an advanced preclinical lead whose value hinges on concurrent progress in pharmaceutical formulation to enable reliable systemic delivery and thorough investigation of its selectivity and safety profile in relevant *in vivo* models.

A range of terpenoids—including Oleanolic Acid, Oridonin, Cucurbitacin B, Dihydroartemisinin, and Costunolide—demonstrate the ability to counteract EGFR TKI resistance through distinct mechanisms in preclinical NSCLC models. These include direct pathway inhibition (e.g., PI3K-Akt-mTOR suppression by Oleanolic Acid), multi-target blockade (e.g., concurrent MEK1 and Akt1/2 inhibition by Costunolide), and induction of alternative cell death modalities such as ferroptosis by Dihydroartemisinin (Silva et al., 2015; Yan et al., 2018; Gao et al., 2024; Zhou et al., 2024; Chen et al., 2019a; Xiao et al., 2016; Khan et al., 2017; Liu et al., 2019). While this group of compounds exemplifies the rich mechanistic diversity within terpenoids, their collective translation is hindered by shared and compound-specific challenges. A principal barrier is the typically unfavorable pharmacokinetic profile common to many terpenoids, characterized by poor solubility, rapid metabolism, and limited bioavailability. With the exception of the semi-synthetic artemisinin derivative Dihydroartemisinin, most remain far from clinical evaluation for NSCLC. Furthermore, strategies like ferroptosis induction or dual kinase inhibition, though mechanistically novel, carry undefined risks of off-target toxicity and systemic metabolic disruption that have not been adequately assessed (Cai et al., 2021; Lai et al., 2023; Tian et al., 2022). Therefore, these compounds largely represent early-stage chemical probes that illuminate resistance biology; their advancement into therapeutics would require substantial medicinal chemistry optimization and comprehensive preclinical safety studies tailored to their specific mechanisms.

### 4.3 Flavonoids

Quercetin demonstrates a broad-spectrum, multi-target approach to overcoming EGFR TKI resistance in preclinical models. It directly inhibits EGFR phosphorylation and downstream ERK/MEK and Akt/S6K pathways, induces autophagy-dependent cell death, and concurrently blocks key bypass receptors such as c-Met, Her-2, AXL, and IGF1R (Ge et al., 2023; Chan et al., 2023; Jiao et al., 2023; Alam et al., 2022). This multi-pronged action translates to synergistic cytotoxicity and apoptosis with erlotinib in resistant cells (Ramirez et al., 2021). To address its primary pharmacokinetic flaw of extremely low bioavailability, advanced formulations like anti-EGFR antibody-conjugated nanovesicles have been developed, which improve targeting and efficacy in animal models (Huang et al., 2021; Cui et al., 2022). The derivative hyperoside also shows activity against T790M-positive cells via the FoxO1/CCAT1 axis (Hu Z. et al., 2020). Quercetin epitomizes the classic challenge in natural product drug development: potent and mechanistically diverse *in vitro* activity that is rendered almost irrelevant by abysmal pharmacokinetic properties. Its well-documented negligible oral bioavailability and rapid systemic clearance mean that the effective concentrations used in cellular studies are pharmacologically unachievable in humans via conventional administration. The sophisticated nano-formulations reported are not mere improvements but essential prerequisites for any serious therapeutic consideration, yet they remain in early preclinical testing (Huang et al., 2021; Cui et al., 2022). Furthermore, its inhibition of a wide array of RTKs, while beneficial for overcoming resistance, significantly increases the risk of unpredictable off-target effects and drug-drug interactions (Alam et al., 2022). Therefore, quercetin serves as a compelling proof-of-concept molecule for multi-target therapy, but its future lies not in the compound itself, but in the successful clinical translation of the advanced delivery technologies designed to rescue it.

Luteolin demonstrates a multi-modal mechanism against EGFR TKI resistance in preclinical NSCLC models. It directly binds to and inhibits EGFR activation, blocking downstream ERK/MEK and Akt/mTOR pathways, while also modulating apoptosis regulators and inducing autophagy (Zhang J. et al., 2024; Zhang et al., 2022; Ambrose et al., 2018; Hong et al., 2014; Huang G. et al., 2023). These actions contribute to its synergistic effects with erlotinib in resistant cells (Ganthala et al., 2022). Computational studies support its direct interaction with EGFR, and network pharmacology analyses suggest broader effects on pathways like PI3K-Akt/MDM2-p53 (Jiao et al., 2023; Maiti et al., 2021; Yi et al., 2022; Ye et al., 2023). Despite its promising multi-target profile *in vitro*, luteolin’s therapeutic potential is critically limited by its extremely poor bioavailability, a characteristic flaw of many flavonoids. The effective concentrations used in mechanistic studies are pharmacologically unachievable with conventional oral dosing due to rapid metabolism and clearance. While its binding to EGFR is computationally validated, this interaction must be confirmed in physiologically relevant systems that account for its pharmacokinetic behavior. Furthermore, its broad effects on multiple core pathways increase the risk of unpredictable biological consequences and potential toxicity when combined with TKIs (Yi et al., 2022; Ye et al., 2023). Therefore, luteolin primarily serves as a valuable tool compound for validating multi-target inhibition strategies; its development as a drug requires prior solutions to its inherent delivery challenges through advanced formulation or prodrug strategies.

Apigenin demonstrates a multi-target mechanism against EGFR TKI resistant NSCLC in preclinical models. It inhibits EGFR phosphorylation and downstream PI3K/Akt and MAPK pathways, while also suppressing EMT mediators like Snail/Slug (Chang et al., 2018). In combination with gefitinib, apigenin disrupts metabolic (HIF-1α, c-Myc) and autophagic adaptations in L858R/T790M-mutant cells to induce apoptosis (Chen et al., 2019b). As a key component of the compound Feiyanning, it has also been shown to reverse resistance by targeting the IGF1R-PI3K-Akt axis (Han et al., 2025). Apigenin’s promising preclinical synergy is overshadowed by the severe pharmacokinetic limitations common to its flavonoid class, including negligible oral bioavailability and rapid systemic elimination. The effective concentrations required to observe its multi-pathway effects *in vitro* are unlikely to be achieved or sustained in human tumors. Furthermore, its activity against targets like IGF1R, while mechanistically valuable for overcoming resistance, expands its pharmacological footprint and raises concerns about off-target effects and an increased risk of drug-drug interactions in a clinical combination setting (Han et al., 2025). Therefore, apigenin exemplifies a compound whose translational feasibility is currently low, serving more as a mechanistic blueprint for multi-pathway inhibition than as an immediate drug candidate. Its potential utility would depend on the development of formulations that dramatically enhance its bioavailability and stability.

Dihydromyricetin (DHM) and hydroxygenkwanin (HGK) represent flavonoids that target EGFR stability to overcome resistance. DHM promotes the ubiquitin-mediated degradation of survivin, suppressing signaling from both wild-type and mutant EGFR (Li et al., 2023). HGK induces apoptosis by promoting EGFR ubiquitination and degradation, concurrently inhibiting downstream STAT3, Akt, and ERK pathways, with transcriptomic data supporting its role in modulating ubiquitination networks (Leu et al., 2020). Preclinical models report antitumor activity for both compounds. While the mechanism of inducing EGFR degradation is pharmacologically attractive, the development of DHM and HGK is constrained by the same critical limitations that plague most flavonoids: very poor bioavailability and uncharacterized human pharmacokinetics. Claims of “low cytotoxicity to normal cells” are based on limited *in vitro* assays and do not constitute an adequate safety profile for therapeutic development. Promoting protein degradation via the ubiquitin-proteasome system, as both compounds appear to do, carries a non-specific risk of disrupting the degradation of essential cellular proteins, a potential toxicity that has not been evaluated. Therefore, DHM and HGK are early-stage tool compounds that validate EGFR degradation as a resistance strategy; their advancement would require extensive medicinal chemistry to improve drug-like properties and thorough investigations into degradation selectivity and systemic safety.

### 4.4 Polyphenols

Curcumin, extensively studied for its broad multi-target activity, shows preclinical promise in countering EGFR TKI resistance by inhibiting EGFR phosphorylation, downstream ERK/MEK and Akt/S6K pathways, and concurrently blocking bypass receptors such as c-Met, Her-2, and AXL (Ye et al., 2012). These mechanisms contribute to its synergistic induction of apoptosis and autophagy-dependent cell death in combination with erlotinib in resistant models (Chen P. et al., 2019; Zhang et al., 2019; Li et al., 2013). To overcome its well-documented pharmacokinetic deficiencies—negligible oral bioavailability, rapid metabolism, and poor stability—research has advanced along two parallel paths: the development of synthetic analogs and hybrids (e.g., WZ35, CP compounds) with enhanced potency and stability, and the design of sophisticated nano-delivery systems (e.g., antibody-conjugated nanovesicles) that improve targeted delivery and *in vivo* efficacy (Chen P. et al., 2019; Zhang et al., 2019; Li et al., 2013). Computational studies further support its direct binding to EGFR mutants, and additional mechanisms such as Ca^2+^/calmodulin-mediated EGFR degradation have been identified (Rajeeve et al., 2025; Li et al., 2025). Curcumin exemplifies a natural compound whose compelling multi-target pharmacology *in vitro* is fundamentally disconnected from clinical feasibility due to intrinsic pharmacokinetic failures. Consequently, the translational focus has shifted decisively from the native molecule toward second-generation engineered analogs and advanced delivery platforms that aim to preserve its polypharmacological logic while conferring drug-like properties. Claims regarding its safety profile in animal modelsare thus largely incidental, as the parent compound cannot achieve pharmacologically relevant exposures in humans (Wang S. et al., 2025). The future of curcumin-based intervention in NSCLC therefore rests not on curcumin itself, but on these rationally designed successors that seek to transform its mechanistic promise into a therapeutically viable entity.

Resveratrol demonstrates multi-modal activity against EGFR TKI resistance in preclinical NSCLC models. It directly inhibits EGFR phosphorylation and downstream ERK/MEK and Akt/mTOR pathways, while also modulating cell death and senescence through calcium-mediated SERCA inhibition, ER stress, and AMPK activation (Li W. et al., 2016; Zhu et al., 2015; Lu et al., 2019). These actions contribute to its synergistic effects with gefitinib, partly by inhibiting drug efflux transporters. To overcome its poor bioavailability, derivatives with improved potency (e.g., TMS, DMU-212, YI-12) have been developed, as well as nano-formulations for co-delivery with chemotherapy (Zhao et al., 2023; Soonthonsrima et al., 2025; Song et al., 2018). Despite its well-characterized multi-target pharmacology, resveratrol’s therapeutic potential is critically constrained by its unfavorable pharmacokinetics, including low oral bioavailability, rapid conjugation, and short half-life. The promising activities observed *in vitro* and in animal models often employ concentrations or formulations not attainable in humans via conventional administration. The development of more potent derivativesand advanced delivery systemsis a direct and necessary response to these limitations. However, these optimized variants themselves remain in early preclinical stages. Furthermore, resveratrol’s pleiotropic effects—such as SERCA inhibition and transporter modulation—while mechanistically valuable, introduce a significant risk of off-target physiological disruption and complex drug interactions. Therefore, resveratrol serves primarily as a pharmacophore for drug design; its clinical translation in oncology depends on the success of its engineered successors in achieving a favorable balance between multi-target efficacy and pharmacokinetic/safety profiles.

Epigallocatechin gallate (EGCG) is a well-characterized polyphenol that directly targets the EGFR ATP-binding site, leading to receptor internalization and degradation, and suppression of downstream MAPK, PI3K/Akt, and STAT3 pathways (Minnelli et al., 2020; Meng et al., 2019). It inhibits proliferation, induces apoptosis, and shows anti-metastatic activity across multiple NSCLC cell lines (Minnelli et al., 2021; Polonio-Alcalá et al., 2025). EGCG synergizes with EGFR-TKIs and antibodies to overcome resistance, and derivatives such as PBOG, EGCG-erlotinib conjugates have been designed for improved targeting (Wang J. et al., 2019; Zhou et al., 2023; Sun et al., 2022; Zi et al., 2025). Computational studies support its binding to EGFR and guide analog design (Alam et al., 2022; Bommu et al., 2019). Despite its clear mechanistic rationale and promising preclinical data, EGCG’s clinical development for NSCLC faces substantial hurdles. Its low oral bioavailability, extensive metabolism, and instability at physiological pH severely limit the achievable systemic concentrations, making the effective doses used in many *in vitro* studies pharmacologically irrelevant in humans. While combination studies in mice show promise, these models often use non-physiological administration routes or doses (Meng et al., 2019; Huang Y. et al., 2023). The reported “favorable safety profile” in limited trials pertains to its consumption as a dietary component, not to the high-dose, targeted therapeutic regimens required for oncology, where toxicity concerns (e.g., hepatotoxicity at high doses) emerge. Furthermore, its direct EGFR binding may be compromised by common resistance mutations (Minnelli et al., 2020). Therefore, EGCG currently serves as a lead compound demonstrating the feasibility of natural product-based EGFR inhibition; its therapeutic future likely depends on the development of bioavailable analogs or prodrugs that overcome its pharmacokinetic shortcomings while maintaining target specificity.

Gallic acid (GA) demonstrates a multi-mechanistic profile against EGFR TKI resistance, including direct inhibition of EGFR phosphorylation and downstream PI3K/Akt and RAS/ERK pathways, specific blockade of Src-Stat3 signaling, and promotion of EGFR degradation via the proteasome in mutant cells (Kang et al., 2020; Wang and Bao, 2020; Phan et al., 2016; Nam et al., 2016). It also modulates immune-related pathways by upregulating p53/miR-34a and downregulating PD-L1 (Kang et al., 2020). Preclinical xenograft models support its antitumor efficacy (Phan et al., 2016). While GA’s diverse mechanisms are pharmacologically appealing, its development is constrained by incomplete pharmacokinetic characterization and unaddressed delivery challenges. As a small phenolic acid, its absorption, distribution, metabolism, and excretion (ADME) properties in the context of cancer therapy are poorly defined, making it unclear whether effective *in vivo* concentrations can be achieved. Furthermore, its activity in promoting EGFR degradation and modulating PD-L1, while mechanistically synergistic, increases the risk of unintended biological effects and potential immune-related adverse events that have not been evaluated (Zhou et al., 2025; Nam et al., 2016). Therefore, GA remains an early-stage compound whose translational potential depends on comprehensive ADME studies, safety pharmacology assessments, and potentially formulation strategies to ensure targeted delivery and minimize systemic toxicity.

Ellagic acid (EA) shows a distinct profile in countering EGFR TKI resistance. It exhibits strong binding affinity for EGFR, reportedly exceeding some standard therapies, to directly inhibit its activity (Ayaz et al., 2022). Notably, EA displays selective anti-proliferative effects against TKI-resistant NSCLC cells while sparing sensitive ones. Its combination with erlotinib demonstrates synergistic antitumor activity in both cellular and xenograft models of EGFR-mutant NSCLC (Xie C. et al., 2020). EA’s promising selectivity and synergy *in vitro* are offset by significant and poorly characterized pharmacokinetic barriers. As a high-molecular-weight polyphenol, it is expected to have very low oral bioavailability, and its absorption, metabolism, and tissue distribution in the context of cancer treatment have not been systematically studied. The claim of superior binding affinity requires validation in physiologically relevant systems (Ayaz et al., 2022). Furthermore, its selective activity against resistant cells, while intriguing, raises questions about the underlying mechanism and whether this selectivity translates to a therapeutic window *in vivo*, or if it merely reflects differential baseline metabolic or uptake properties (Xie C. et al., 2020). Therefore, EA represents an interesting but early-stage candidate whose development necessitates thorough pharmacokinetic investigation and mechanistic deconvolution of its observed selectivity before its adjuvant potential can be realistically assessed.

### 4.5 Others

Shikonin (a naphthoquinone) and silibinin (a flavonolignan), along with other compounds like xanthohumol, represent diverse chemical classes with activity against EGFR TKI resistance. Shikonin induces ROS-mediated apoptosis and EGFR degradation, showing synergy with TKIs in both mutant and wild-type EGFR contexts (Li et al., 2017; Hsieh et al., 2017; Hu X. et al., 2020; Tang et al., 2018; Li B. et al., 2018; Li Y. L. et al., 2018). Silibinin inhibits EGFR downstream pathways (PI3K/Akt, JAK/STAT) and reverses EMT by modulating specific microRNAs, with its bioavailability improved by formulation as silibinin-meglumine (Wang, 2025; Rugamba et al., 2021; Hou et al., 2018; Cufí et al., 2013a; Cufí et al., 2013b; Corominas-Faja et al., 2013). Other agents, such as xanthohumol, target bypass resistance by promoting Ets-1 degradation to disrupt c-Met signaling (Ma et al., 2024). The promising multi-target activities of these compounds are counterbalanced by significant and often unaddressed developmental challenges. For shikonin, its potent ROS induction is a double-edged sword, posing a clear risk of off-target toxicity and oxidative damage to normal tissues—a safety concern that has not been adequately evaluated in preclinical models. While silibinin-meglumine addresses solubility, the broader pharmacokinetic profile, long-term safety, and optimal dosing for combination therapy with TKIs remain undefined (Corominas-Faja et al., 2013). Many of the other mentioned compounds such as psorachromene and genipin derivatives are at an early proof-of-concept stage with no available data on absorption, metabolism, or toxicity (Wang et al., 2022; Kim et al., 2025). Therefore, this group of compounds collectively illustrates a spectrum of interesting mechanistic leads; however, their progression beyond basic research requires dedicated investigation into their drug-like properties, selectivity, and safety, moving from phenotypic observations to thorough translational pharmacology.

## 5 Discussion

The extensive preclinical evidence synthesized in this review underscores the compelling rationale for exploring natural compounds as strategic partners to overcome EGFR TKI resistance in NSCLC. Their inherent polypharmacology enables the simultaneous targeting of the core EGFR pathway and its key resistance-associated bypass tracks, a theoretical advantage over sequential single-target therapies. However, the translation of this mechanistic promise into clinical practice is fraught with systemic challenges. This discussion moves beyond reiterating mechanisms to critically evaluate the primary translational barriers and propose a strategic roadmap for future development.

### 5.1 The translational chasm: systemic barriers beyond efficacy

The principal obstacle to clinical translation lies not in a lack of *in vitro* efficacy, but in a pervasive disconnect between promising cellular activity and viable drug-like properties. Most compounds reviewed—particularly flavonoids, polyphenols, and many terpenoids—suffer from extremely poor oral bioavailability, owing to low aqueous solubility, rapid phase II metabolism, and swift systemic clearance (Smeu et al., 2025). Consequently, effective concentrations demonstrated in cell culture are often pharmacologically unattainable in human plasma using the native compounds, rendering much reported *in vitro* synergy clinically irrelevant without deliberate pharmaceutical intervention. Furthermore, claims of “favorable safety profiles” are typically based on limited acute toxicity assays or historical use, rather than rigorous, oncology-focused toxicology studies. Critical gaps persist concerning chronic toxicity, organ-specific liabilities such as hepatotoxicity from high-dose EGCG or celastrol, and mechanism-based risks—including off-target oxidative damage induced by ROS-promoting agents like shikonin and dihydroartemisinin, or disruption of the ubiquitin-proteasome system by compounds that enhance protein degradation. Additionally, the potential for drug-drug interactions with concurrently administered TKIs, often mediated through CYP450 enzyme modulation, remains largely uncharacterized. Beyond these scientific hurdles, the development of complex natural products also faces unique regulatory challenges, including batch-to-batch variability, precise quantification of active constituents, and demonstration of pharmacokinetic reproducibility—complications that are exacerbated when the active moiety includes metabolites or multiple synergistic components.

### 5.2 Strategic pathways to clinical translation

To bridge this translational chasm, future research must pivot decisively from descriptive mechanism-finding to problem-solving engineering and rigorous clinical science. Overcoming pharmacokinetic limitations is non-optional and should be prioritized through two main engineering strategies. First, the development of advanced drug delivery systems, particularly nanotechnology-based platforms such as ligand-targeted nanoparticles and exosomes, is essential. These systems can enhance solubility, protect compounds from metabolism, promote tumor-selective delivery, and enable the controlled co-delivery of natural compound-TKI combinations, as evidenced in preliminary studies with celastrol and Rg3. Second, medicinal chemistry-driven optimization represents a more direct developmental path, involving the creation of synthetic analogs or prodrugs (e.g., curcumin hybrid CP, resveratrol derivative TMS) that retain the core multi-target pharmacophore while significantly improving chemical stability, potency, and overall pharmacokinetic properties.

Concurrently, the field must generate robust, translational evidence beyond model system observations. This necessitates hierarchical preclinical validation using pharmacologically relevant models—such as a “resistance model repository” of patient-derived organoids and xenografts—that employ the intended clinical formulations. Furthermore, early-phase trials must be meticulously designed with the primary objectives of establishing the pharmacokinetic profile and maximum tolerated dose of novel formulations in combination with standard TKIs. Promising candidates with preliminary clinical signals, like Ginsenoside Rg3, warrant prospective, biomarker-driven phase I/II trials to formally assess combination safety and efficacy.

Finally, leveraging enabling technologies is crucial for precise target and patient selection. Artificial intelligence and computational tools can accelerate the identification and ADMET prediction of natural product-inspired leads, guiding rational design. Complementing this, the development of mechanistic biomarkers—such as specific mutation profiles or expression levels of bypass proteins—is vital for enriching clinical trials with patients most likely to benefit from a given natural compound combination, steering the field towards a more personalized therapeutic approach.

### 5.3 Future perspective

Natural compounds offer a rich repository of molecular templates for designing multi-targeted interventions against the complex network of EGFR TKI resistance. Their future in NSCLC therapy, however, does not lie in their direct application as dietary supplements or crude extracts. The path forward requires a strategic convergence: integrating the nuanced biological insights gained from natural products with the precision-driven disciplines of medicinal chemistry, advanced drug delivery, and rigorous clinical oncology. Ultimate success will be measured by the development of engineered therapeutic agents—whether as optimized synthetic analogs or sophisticated nano-formulations—capable of reliably translating multi-target potential into clinical benefit for patients, thereby expanding the therapeutic arsenal against EGFR-TKI-resistant NSCLC.

## 6 Conclusion

This review establishes that natural compounds, by virtue of their inherent multi-target capabilities, represent a unique strategic avenue to overcome the complex challenge of EGFR TKI resistance in NSCLC. Their ability to simultaneously modulate the core EGFR axis and key resistance pathways provides a strong rationale for their development as sensitizing agents in rational combination therapies. However, this significant preclinical promise must now be bridged to clinical reality through rigorous pharmaceutical optimization and dedicated clinical trials. Therefore, the future of this field lies in translating these multifaceted mechanistic insights into clinically validated, next-generation combination regimens for the benefit of patients.

## References

[B1] Alam M. Alam S. Shamsi A. Adnan M. Elasbali A. M. Al-Soud W. A. (2022). Bax/Bcl-2 Cascade is regulated by the EGFR pathway: therapeutic targeting of non-small cell lung cancer. Front. Oncol. 12, 869672. 10.3389/fonc.2022.869672 35402265 PMC8990771

[B2] Ambrose G. O. Afees O. J. Nwamaka N. C. Simon N. Oluwaseun A. A. Soyinka T. (2018). Selection of luteolin as a potential antagonist from molecular docking analysis of EGFR mutant. Bioinformation 14 (5), 241–247. 10.6026/97320630014241 30108422 PMC6077815

[B3] Andrés C. M. C. Pérez D. L. Lastra J. M. Bustamante Munguira E. Pérez-Lebeña E. (2024). Michael acceptors as anti-cancer compounds: coincidence or causality? Int. J. Mol. Sci. 25 (11), 6099. 10.3390/ijms25116099 38892287 PMC11172677

[B4] Ayaz Z. Zainab B. Rashid U. Darwish N. M. Gatasheh M. K. Abbasi A. M. (2022). *In silico* screening of synthetic and natural compounds to inhibit the binding capacity of heavy metal compounds against EGFR protein of lung cancer. BioMed Res. Int. 2022, 2941962. 10.1155/2022/2941962 35607306 PMC9124118

[B5] Bellevicine C. Malapelle U. De Luca C. Iaccarino A. Troncone G. (2014). EGFR analysis: current evidence and future directions. Diagn. Cytopathol. 42 (11), 984–992. 10.1002/dc.23142 24619906

[B6] Bommu U. D. Konidala K. K. Pabbaraju N. Yeguvapalli S. (2019). QSAR modeling, pharmacophore-based virtual screening, and ensemble docking insights into predicting potential epigallocatechin gallate (EGCG) analogs against epidermal growth factor receptor. J. Recept. Signal Transduct. Res. 39 (1), 18–27. 10.1080/10799893.2018.1564151 31223050

[B7] Bouchard N. Daaboul N. (2025). Lung cancer: targeted therapy in 2025. Curr. Oncol. Tor. Ont. 32 (3), 146. 10.3390/curroncol32030146 40136350 PMC11941068

[B8] Breindel J. L. Haskins J. W. Cowell E. P. Zhao M. Nguyen D. X. Stern D. F. (2013). EGF receptor activates MET through MAP kinases to enhance non-small cell lung carcinoma invasion and brain metastasis. Cancer Research 73 (16), 5053–5065. 10.1158/0008-5472.CAN-12-3775 23794705 PMC3745527

[B9] Cai X. Miao J. Sun R. Wang S. Molina-Vila M. A. Chaib I. (2021). Dihydroartemisinin overcomes the resistance to osimertinib in EGFR-Mutant non-small-cell lung cancer. Pharmacol. Res. 170, 105701. 10.1016/j.phrs.2021.105701 34087353

[B10] Castellanos E. Feld E. Horn L. (2017). Driven by mutations: the predictive value of mutation subtype in EGFR-mutated non-small cell lung cancer. J. Thorac. Oncol. Official Publ. Int. Assoc. Study Lung Cancer 12 (4), 612–623. 10.1016/j.jtho.2016.12.014 28017789

[B11] Chan C. Y. Hong S. C. Chang C. M. Chen Y. H. Liao P. C. Huang C. Y. (2023). Oral squamous cell carcinoma cells with acquired resistance to erlotinib are sensitive to anti-cancer effect of quercetin via pyruvate kinase M2 (PKM2). Cells 12 (1), 179. 10.3390/cells12010179 36611972 PMC9818869

[B12] Chang J. H. Cheng C. W. Yang Y. C. Chen W. S. Hung W. Y. Chow J. M. (2018). Downregulating CD26/DPPIV by apigenin modulates the interplay between Akt and snail/slug signaling to restrain metastasis of lung cancer with multiple EGFR statuses. J. Experimental and Clinical Cancer Research CR 37 (1), 199. 10.1186/s13046-018-0869-1 30134935 PMC6104010

[B13] Chen S. F. Zhang Z. Y. Zhang J. L. (2017). Matrine increases the inhibitory effects of afatinib on H1975 cells via the IL-6/JAK1/STAT3 signaling pathway. Mol. Med. Rep. 16 (3), 2733–2739. 10.3892/mmr.2017.6865 28656237 PMC5547971

[B14] Chen P. Huang H. P. Wang Y. Jin J. Long W. G. Chen K. (2019). Curcumin overcome primary gefitinib resistance in non-small-cell lung cancer cells through inducing autophagy-related cell death. J. Experimental and Clinical Cancer Research CR 38 (1), 254. 10.1186/s13046-019-1234-8 31196210 PMC6567416

[B15] Chen Z. Huang K. Y. Ling Y. Goto M. Duan H. Q. Tong X. H. (2019a). Discovery of an oleanolic Acid/hederagenin-nitric oxide donor hybrid as an EGFR tyrosine kinase inhibitor for non-small-cell lung cancer. J. Nat. Prod. 82 (11), 3065–3073. 10.1021/acs.jnatprod.9b00659 31718182

[B16] Chen Z. Tian D. Liao X. Zhang Y. Xiao J. Chen W. (2019b). Apigenin combined with gefitinib blocks autophagy flux and induces apoptotic cell death through inhibition of HIF-1α, c-Myc, p-EGFR, and glucose metabolism in EGFR L858R+T790M-Mutated H1975 cells. Front. Pharmacol. 10, 260. 10.3389/fphar.2019.00260 30967777 PMC6438929

[B17] Chen P. Dai C. H. Shi Z. H. Wang Y. Wu J. N. Chen K. (2021). Synergistic inhibitory effect of berberine and icotinib on non-small cell lung cancer cells via inducing autophagic cell death and apoptosis. Apoptosis. Int. J. Program. Cell Death 26 (11-12), 639–656. 10.1007/s10495-021-01694-w 34743246

[B18] Chen Z. Vallega K. A. Chen H. Zhou J. Ramalingam S. S. Sun S. Y. (2022). The natural product berberine synergizes with osimertinib preferentially against MET-amplified osimertinib-resistant lung cancer via direct MET inhibition. Pharmacol. Res. 175, 105998. 10.1016/j.phrs.2021.105998 34826601 PMC8755628

[B19] Cheng H. Ge X. Zhuo S. Gao Y. Zhu B. Zhang J. (2018). β-Elemene synergizes with gefitinib to inhibit stem-like phenotypes and progression of lung cancer via down-regulating EZH2. Front. Pharmacol. 9, 1413. 10.3389/fphar.2018.01413 30555330 PMC6284059

[B20] Corominas-Faja B. Oliveras-Ferraros C. Cuyàs E. Segura-Carretero A. Joven J. Martin-Castillo B. (2013). Stem cell-like ALDH(bright) cellular states in EGFR-mutant non-small cell lung cancer: a novel mechanism of acquired resistance to erlotinib targetable with the natural polyphenol silibinin. Cell CycleGeorget. Tex. 12 (21), 3390–3404. 10.4161/cc.26417 24047698 PMC3895428

[B21] Cuan X. Yang X. Zhu W. Zhao Y. Luo R. Huang Y. (2023). Antitumor effects of erlotinib in combination with berberine in A431 cells. BMC Pharmacology and Toxicology 24 (1), 29. 10.1186/s40360-023-00661-2 37170144 PMC10173514

[B22] Cufí S. Bonavia R. Vazquez-Martin A. Corominas-Faja B. Oliveras-Ferraros C. Cuyàs E. (2013a). Silibinin meglumine, a water-soluble form of milk thistle silymarin, is an orally active anti-cancer agent that impedes the epithelial-to-mesenchymal transition (EMT) in EGFR-mutant non-small-cell lung carcinoma cells. Food Chem. Toxicol. Int. J. Publ. Br. Industrial Biol. Res. Assoc. 60, 360–368. 10.1016/j.fct.2013.07.063 23916468

[B23] Cufí S. Bonavia R. Vazquez-Martin A. Oliveras-Ferraros C. Corominas-Faja B. Cuyàs E. (2013b). Silibinin suppresses EMT-driven erlotinib resistance by reversing the high miR-21/low miR-200c signature *in vivo* . Sci. Rep. 3, 2459. 10.1038/srep02459 23963283 PMC3748425

[B24] Cui X. Zhang F. Zhao Y. Li P. Wang T. Xu Z. (2022). A novel ligand-modified nanocomposite microparticles improved efficiency of quercetin and paclitaxel delivery in the non-small cell lung cancer. Drug Deliv. 29 (1), 3123–3133. 10.1080/10717544.2022.2120567 36151722 PMC9848416

[B25] Dai C. H. Zhu L. R. Wang Y. Tang X. P. Du Y. J. Chen Y. C. (2021). Celastrol acts synergistically with afatinib to suppress non-small cell lung cancer cell proliferation by inducing paraptosis. J. Cell. Physiology 236 (6), 4538–4554. 10.1002/jcp.30172 33230821

[B26] Fan X. X. Li N. Wu J. L. Zhou Y. L. He J. X. Liu L. (2014). Celastrol induces apoptosis in gefitinib-resistant non-small cell lung cancer cells via caspases-dependent pathways and Hsp90 client protein degradation. Mol. Basel, Switz. 19 (3), 3508–3522. 10.3390/molecules19033508 24662070 PMC6271537

[B27] Fan X. X. Yao X. J. Xu S. W. Wong V. K. W. He J. X. Ding J. (2015). (Z)3,4,5,4’-trans-tetramethoxystilbene, a new analogue of resveratrol, inhibits gefitinb-resistant non-small cell lung cancer via selectively elevating intracellular calcium level. Sci. Rep. 5, 16348. 10.1038/srep16348 26542098 PMC4635386

[B28] Fu S. Li G. Zang W. Zhou X. Shi K. Zhai Y. (2022). Pure drug nano-assemblies: a facile carrier-free nanoplatform for efficient cancer therapy. Acta Pharm. Sin. B 12 (1), 92–106. 10.1016/j.apsb.2021.08.012 35127374 PMC8799886

[B29] Ganthala P. D. Alavala S. Chella N. Andugulapati S. B. Bathini N. B. Sistla R. (2022). Co-encapsulated nanoparticles of erlotinib and quercetin for targeting lung cancer through nuclear EGFR and PI3K/AKT inhibition. Colloids Surfaces. B, Biointerfaces 211, 112305. 10.1016/j.colsurfb.2021.112305 34998178

[B30] Gao K. Chen Z. Zhang N. Jiang P. (2024). High throughput virtual screening and validation of plant-based EGFR L858R kinase inhibitors against non-small cell lung cancer: an integrated approach utilizing GC-MS, network pharmacology, docking, and molecular dynamics. Saudi Pharmaceutical Journal SPJ The Official Publication Saudi Pharm. Soc. 32 (9), 102139. 10.1016/j.jsps.2024.102139 39139718 PMC11318564

[B31] Ge Z. Xu M. Ge Y. Huang G. Chen D. Ye X. (2023). Inhibiting G6PD by quercetin promotes degradation of EGFR T790M mutation. Cell Rep. 42 (11), 113417. 10.1016/j.celrep.2023.113417 37950872

[B32] Gu Y. He H. Qiao S. Shao Y. Wang L. Zhang Z. (2025). EGFR: new insights on its activation and mutation in tumor and tumor immunotherapy. Adv. Sci. 12 (36), e05785. 10.1002/advs.202505785 40859900 PMC12462975

[B33] Han Y. Jiao L. Zhou H. Sang S., A, G. Liu R. (2025). Exploring the mechanism of feiyanning formula and its extract apigenin against EGFR-TKIs resistance in non-small cell lung cancer based on UPLC-HRMS and experimental validation. J. Ethnopharmacol. 351, 120120. 10.1016/j.jep.2025.120120 40499802

[B34] Hong Z. Cao X. Li N. Zhang Y. Lan L. Zhou Y. (2014). Luteolin is effective in the non-small cell lung cancer model with L858R/T790M EGF receptor mutation and erlotinib resistance. Br. J. Pharmacol. 171 (11), 2842–2853. 10.1111/bph.12610 24471765 PMC4243859

[B35] Hou X. Du H. Quan X. Shi L. Zhang Q. Wu Y. (2018). Silibinin inhibits NSCLC metastasis by targeting the EGFR/LOX pathway. Front. Pharmacol. 9, 21. 10.3389/fphar.2018.00021 29472856 PMC5809401

[B36] Hsieh Y. S. Liao C. H. Chen W. S. Pai J. T. Weng M. S. (2017). Shikonin inhibited migration and invasion of human lung cancer cells via suppression of c-Met-Mediated epithelial-to-mesenchymal transition. J. Cell. Biochem. 118 (12), 4639–4651. 10.1002/jcb.26128 28485480

[B37] Hu X. Zhang Z. Y. Wu L. W. Zeng L. H. Chen H. Zhu H. J. (2020). A natural anthraquinone derivative shikonin synergizes with AZD9291 against wtEGFR NSCLC cells through reactive oxygen species-mediated endoplasmic reticulum stress. Phytomedicine Int. J. Phytotherapy Phytopharm. 68, 153189. 10.1016/j.phymed.2020.153189 32070867

[B38] Hu Z. Zhao P. Xu H. (2020). Hyperoside exhibits anticancer activity in non-small cell lung cancer cells with T790M mutations by upregulating FoxO1 via CCAT1. Oncol. Rep. 43 (2), 617–624. 10.3892/or.2019.7440 31894285

[B40] Huang K. Y. Wang T. H. Chen C. C. Leu Y. L. Li H. J. Jhong C. L. (2021). Growth suppression in lung cancer cells harboring EGFR-C797S mutation by quercetin. Biomolecules 11 (9), 1271. 10.3390/biom11091271 34572484 PMC8470952

[B41] Huang G. Liu X. Jiang T. Cao Y. Sang M. Song X. (2023). Luteolin overcomes acquired resistance to osimertinib in non-small cell lung cancer cells by targeting the HGF-MET-Akt pathway. Am. J. Cancer Res. 13 (9), 4145–4162. 37818074 PMC10560942

[B42] Huang Y. Cuan X. Zhu W. Yang X. Zhao Y. Sheng J. (2023). An EGCG derivative in combination with nimotuzumab for the treatment of wild-type EGFR NSCLC. Int. J. Mol. Sci. 24 (18), 14012. 10.3390/ijms241814012 37762316 PMC10531337

[B43] Huang Q. Li Y. Huang Y. Wu J. Bao W. Xue C. (2025). Advances in molecular pathology and therapy of non-small cell lung cancer. Signal Transduct. Target. Ther. 10, 186. 10.1038/s41392-025-02243-6 40517166 PMC12167388

[B44] Imtiaz I. Schloss J. Bugarcic A. (2025). Interplay between traditional and scientific knowledge: phytoconstituents and their roles in lung and colorectal cancer signaling pathways. Biomolecules 15 (3), 380. 10.3390/biom15030380 40149916 PMC11940637

[B45] Jänne P. A. Yang J. C. H. Kim D. W. Planchard D. Ohe Y. Ramalingam S. S. (2015). AZD9291 in EGFR inhibitor-resistant non-small-cell lung cancer. N. Engl. J. Med. 372 (18), 1689–1699. 10.1056/NEJMoa1411817 25923549

[B46] Jeon H. Wang S. Song J. Gill H. Cheng H. (2025). Update 2025: management of non-small-cell lung cancer. Lung 203 (1), 53. 10.1007/s00408-025-00801-x 40133478 PMC11937135

[B47] Jiang G. Liu X. Zhang D. Diao Z. Yang X. Tan Q. (2025). A hybrid protein-oxygen nanomedicine overcomes osimertinib resistance in NSCLC via HIF-1α/VEGF/EGFR inhibition. Int. J. Nanomedicine 20, 10389–10405. 10.2147/IJN.S531571 40901248 PMC12400113

[B48] Jiao P. Wang Y. Ren G. Chu D. Li Y. Sang T. (2023). Integrating network pharmacology and experimental validation to elucidate the mechanism of yiqi yangyin decoction in suppressing non-small-cell lung cancer. BioMed Res. Int. 2023, 4967544. 10.1155/2023/4967544 36874921 PMC9980286

[B49] Kang D. Y. Sp N. Jo E. S. Rugamba A. Hong D. Y. Lee H. G. (2020). The inhibitory mechanisms of tumor PD-L1 expression by natural bioactive gallic acid in non-small-cell lung cancer (NSCLC) cells. Cancers 12 (3), 727. 10.3390/cancers12030727 32204508 PMC7140102

[B50] Kang D. Y. Sp N. Lee J. M. Jang K. J. (2021). Antitumor effects of ursolic acid through mediating the inhibition of STAT3/PD-L1 signaling in non-small cell lung cancer cells. Biomedicines 9 (3), 297. 10.3390/biomedicines9030297 33805840 PMC7998465

[B51] Khan N. Jajeh F. Khan M. I. Mukhtar E. Shabana S. M. Mukhtar H. (2017). Sestrin-3 modulation is essential for therapeutic efficacy of cucurbitacin B in lung cancer cells. Carcinogenesis 38 (2), 184–195. 10.1093/carcin/bgw124 27881463 PMC6075515

[B52] Kim D. H. Hwang H. H. Hong J. Seo K. Choi J. H. Je J. H. (2025). Genipin derivative induced the apoptosis and inhibited the invasion and migration of A549 cancer cells via regulation of EGFR/JAK1/STAT3 signaling. Bioorg. and Med. Chem. Lett. 128, 130320. 10.1016/j.bmcl.2025.130320 40592362

[B53] Ko J. L. Lin C. H. Chen H. C. Hung W. H. Chien P. J. Chang H. Y. (2018). Effects and mechanisms of betulinic acid on improving EGFR TKI-resistance of lung cancer cells. Environ. Toxicol. 33 (11), 1153–1159. 10.1002/tox.22621 30136359

[B54] Lai X. Y. Shi Y. M. Zhou M. M. (2023). Dihydroartemisinin enhances gefitinib cytotoxicity against lung adenocarcinoma cells by inducing ROS-dependent apoptosis and ferroptosis. Kaohsiung J. Med. Sci. 39 (7), 699–709. 10.1002/kjm2.12684 37057810 PMC11895908

[B55] Lee Y. J. Kim S. Y. Lee C. (2019). Axl is a novel target of celastrol that inhibits cell proliferation and migration, and increases the cytotoxicity of gefitinib in EGFR mutant non-small cell lung cancer cells. Mol. Med. Rep. 19 (4), 3230–3236. 10.3892/mmr.2019.9957 30816529

[B56] Leu Y. L. Wang T. H. Wu C. C. Huang K. Y. Jiang Y. W. Hsu Y. C. (2020). Hydroxygenkwanin suppresses non-small cell lung cancer progression by enhancing EGFR degradation. Mol. Basel, Switz. 25 (4), 941. 10.3390/molecules25040941 32093124 PMC7070862

[B57] Leung E. L. H. Fan X. X. Wong M. P. Jiang Z. H. Liu Z. Q. Yao X. J. (2016). Targeting tyrosine kinase inhibitor-resistant non-small cell lung cancer by inducing epidermal growth factor receptor degradation via methionine 790 oxidation. Antioxidants and Redox Signal. 24 (5), 263–279. 10.1089/ars.2015.6420 26528827 PMC4753639

[B58] Li S. Liu Z. Zhu F. Fan X. Wu X. Zhao H. (2013). Curcumin lowers erlotinib resistance in non-small cell lung carcinoma cells with mutated EGF receptor. Oncol. Res. 21 (3), 137–144. 10.3727/096504013X13832473330032 24512728

[B59] Li Y. Wang Y. Niu K. Chen X. Xia L. Lu D. (2016). Clinical benefit from EGFR-TKI plus ginsenoside Rg3 in patients with advanced non-small cell lung cancer harboring EGFR active mutation. Oncotarget 7 (43), 70535–70545. 10.18632/oncotarget.12059 27655708 PMC5342572

[B60] Li W. Ma X. Li N. Liu H. Dong Q. Zhang J. (2016). Resveratrol inhibits hexokinases II mediated glycolysis in non-small cell lung cancer via targeting Akt signaling pathway. Exp. Cell Res. 349 (2), 320–327. 10.1016/j.yexcr.2016.11.002 27829129

[B61] Li X. Fan X. X. Jiang Z. B. Loo W. T. Yao X. J. Leung E. L. H. (2017). Shikonin inhibits gefitinib-resistant non-small cell lung cancer by inhibiting TrxR and activating the EGFR proteasomal degradation pathway. Pharmacol. Res. 115, 45–55. 10.1016/j.phrs.2016.11.011 27864022

[B62] Li B. Yuan Z. Jiang J. Rao Y. (2018). Anti-tumor activity of shikonin against afatinib resistant non-small cell lung cancer via negative regulation of PI3K/Akt signaling pathway. Biosci. Rep. 38 (6), BSR20181693. 10.1042/BSR20181693 30420490 PMC6294622

[B63] Li W. Yu X. Tan S. Liu W. Zhou L. Liu H. (2018). Oxymatrine inhibits non-small cell lung cancer via suppression of EGFR signaling pathway. Cancer Med. 7 (1), 208–218. 10.1002/cam4.1269 29239135 PMC5773973

[B64] Li Y. L. Hu X. Li Q. Y. Wang F. Zhang B. Ding K. (2018). Shikonin sensitizes wild-type EGFR NSCLC cells to erlotinib and gefitinib therapy. Mol. Med. Rep. 18 (4), 3882–3890. 10.3892/mmr.2018.9347 30106133 PMC6131653

[B65] Li X. Zhou L. Wang R. Zhang Y. Li W. (2023). Dihydromyricetin suppresses tumor growth via downregulation of the EGFR/Akt/survivin signaling pathway. J. Biochem. Mol. Toxicol. 37 (6), e23328. 10.1002/jbt.23328 36807944

[B66] Li L. Lian X. Ding L. Guo R. Xu J. Bai R. (2025). Calcium triggers calmodulin degradation to induce EGF receptor instability and overcome non-small cell lung cancer resistance to tyrosine kinase inhibitors. J. Biol. Chem. 301 (7), 110305. 10.1016/j.jbc.2025.110305 40447190 PMC12256327

[B67] Liang Y. Zhang T. Jing S. Zuo P. Li T. Wang Y. (2021). 20(S)-Ginsenoside Rg3 inhibits lung cancer cell proliferation by targeting EGFR-mediated Ras/Raf/MEK/ERK pathway. Am. J. Chin. Med. 49 (3), 753–765. 10.1142/S0192415X2150035X 33641655

[B68] Liu P. Xiang Y. Liu X. Zhang T. Yang R. Chen S. (2019). Cucurbitacin B induces the lysosomal degradation of EGFR and suppresses the CIP2A/PP2A/Akt signaling axis in Gefitinib-resistant non-small cell lung cancer. Mol. Basel, Switz. 24 (3), 647. 10.3390/molecules24030647 30759826 PMC6384961

[B69] Liu W. Jiang Z. Wang R. Zhang X. Jiang X. Chen C. (2025). Targeting EGFR-Mcl-1 axis by piperlongumine as a novel strategy for non-small cell lung cancer therapy. Am. J. Chin. Med. 53 (2), 597–619. 10.1142/S0192415X25500235 40145280

[B70] Lu M. Liu B. Xiong H. Wu F. Hu C. Liu P. (2019). Trans-3,5,4´-trimethoxystilbene reduced gefitinib resistance in NSCLCs via suppressing MAPK/Akt/Bcl-2 pathway by upregulation of miR-345 and miR-498. J. Cell. Mol. Med. 23 (4), 2431–2441. 10.1111/jcmm.14086 30701693 PMC6433677

[B71] Lv X. Song Y. Liu T. Zhang D. Ye X. Wang Q. (2025). Ginsenoside Rg3 adjunctively increases the efficacy of gefitinib against NSCLC by regulating EGFR copy number. Pharm. Basel, Switz. 18 (7), 1077. 10.3390/ph18071077 40732366 PMC12300113

[B72] Ma Y. Wang R. Liao J. Guo P. Wang Q. Li W. (2024). Xanthohumol overcomes osimertinib resistance via governing ubiquitination-modulated Ets-1 turnover. Cell Death Discov. 10 (1), 454. 10.1038/s41420-024-02220-y 39468027 PMC11519634

[B73] Maiti P. Nand M. Joshi T. Ramakrishnan M. A. Chandra S. (2021). Identification of luteolin -7-glucoside and epicatechin gallate from vernoniacinerea, as novel EGFR L858R kinase inhibitors against lung cancer: docking and simulation-based study. J. Biomol. Struct. and Dyn. 39 (14), 5048–5057. 10.1080/07391102.2020.1784791 32579072

[B74] Meng J. Chang C. Chen Y. Bi F. Ji C. Liu W. (2019). EGCG overcomes gefitinib resistance by inhibiting autophagy and augmenting cell death through targeting ERK phosphorylation in NSCLC. OncoTargets Ther. 12, 6033–6043. 10.2147/OTT.S209441 31440060 PMC6668247

[B75] Ming H. Lu T. Zhou H. Song W. Dai H. (2024). Synergistic inhibitory effect of atmospheric pressure plasma and berberine on non-small cell lung cancer cells via inducing apoptosis. Mol. Biol. Rep. 52 (1), 37. 10.1007/s11033-024-10132-4 39643828

[B76] Minnelli C. Laudadio E. Mobbili G. Galeazzi R. (2020). Conformational insight on WT- and Mutated-EGFR receptor activation and inhibition by Epigallocatechin-3-Gallate: over a rational basis for the design of selective non-small-cell lung anticancer agents. Int. J. Mol. Sci. 21 (5), 1721. 10.3390/ijms21051721 32138321 PMC7084708

[B77] Minnelli C. Cianfruglia L. Laudadio E. Mobbili G. Galeazzi R. Armeni T. (2021). Effect of Epigallocatechin-3-Gallate on EGFR signaling and migration in non-small cell lung cancer. Int. J. Mol. Sci. 22 (21), 11833. 10.3390/ijms222111833 34769263 PMC8583909

[B78] Modi S. R. Andey T. (2024). Piperlongumine in combination with EGFR tyrosine kinase inhibitors for the treatment of lung cancer cells. Oncol. Res. 32 (11), 1709–1721. 10.32604/or.2024.053972 39449797 PMC11497197

[B79] Murphrey M. B. Quaim L. Rahimi N. Varacallo M. A. (2025). Biochemistry, epidermal growth factor Receptor. Treasure Island (FL): StatPearls Publishing. Available online at: http://www.ncbi.nlm.nih.gov/books/NBK482459/ (Accessed December 1, 2025). 29494066

[B80] Nam B. Rho J. K. Shin D. M. Son J. (2016). Gallic acid induces apoptosis in EGFR-mutant non-small cell lung cancers by accelerating EGFR turnover. Bioorg. and Med. Chem. Lett. 26 (19), 4571–4575. 10.1016/j.bmcl.2016.08.083 27597244

[B81] Phan A. N. H. Hua T. N. M. Kim M. K. Vo V. T. A. Choi J. W. Kim H. W. (2016). Gallic acid inhibition of Src-Stat3 signaling overcomes acquired resistance to EGF receptor tyrosine kinase inhibitors in advanced non-small cell lung cancer. Oncotarget 7 (34), 54702–54713. 10.18632/oncotarget.10581 27419630 PMC5342374

[B82] Polonio-Alcalá E. Ausellé-Bosch S. Riesco-Llach G. Novales P. Feliu L. Planas M. (2025). Elucidating the role of FASN in lung cancer stem cells in sensitive and resistant EGFR-mutated non-small cell lung cancer cells. Lung CancerAuckl. N.Z. 16, 57–72. 10.2147/LCTT.S512936 40452758 PMC12126118

[B83] Rajeeve A. D. Yamuna R. Namboori P. K. K. (2025). Elucidating the potential of EGFR mutated NSCLC and identifying its multitargeted inhibitors. Sci. Rep. 15 (1), 3649. 10.1038/s41598-024-83868-5 39880831 PMC11779874

[B84] Ramirez J. House L. K. Ratain M. J. (2021). Influence of N-acetyltransferase 2 gene polymorphisms on the *in vitro* metabolism of the epidermal growth factor receptor inhibitor rociletinib. Br. J. Clin. Pharmacol. 87 (11), 4313–4322. 10.1111/bcp.14848 33818816

[B85] Reznik T. E. Sang Y. Ma Y. Abounader R. Rosen E. M. Xia S. (2008). Transcription-dependent epidermal growth factor receptor activation by hepatocyte growth factor. Mol. Cancer Research MCR 6 (1), 139–150. 10.1158/1541-7786.MCR-07-0236 18234969 PMC2839502

[B86] Riely G. J. Wood D. E. Ettinger D. S. Aisner D. L. Akerley W. Bauman J. R. (2024). Non-small cell lung cancer, version 4.2024, NCCN clinical practice guidelines in oncology. J. Natl. Compr. Cancer Netw. JNCCN 22 (4), 249–274. 10.6004/jnccn.2204.0023 38754467

[B87] Rossi A. Galetta D. (2022). Systemic therapy for oligoprogression in patients with metastatic NSCLC harboring activating EGFR mutations. Cancers 14 (3), 832. 10.3390/cancers14030832 35159099 PMC8834352

[B88] Rugamba A. Kang D. Y. Sp N. Jo E. S. Lee J. M. Bae S. W. (2021). Silibinin regulates tumor progression and tumorsphere formation by suppressing PD-L1 expression in non-small cell lung cancer (NSCLC) cells. Cells 10 (7), 1632. 10.3390/cells10071632 34209829 PMC8307196

[B89] Saini N. Grewal A. S. Lather V. Gahlawat S. K. (2022). Natural alkaloids targeting EGFR in non-small cell lung cancer: molecular docking and ADMET predictions. Chemico-Biological Interact. 358, 109901. 10.1016/j.cbi.2022.109901 35341731

[B90] Salih D. J. Reiners K. S. Alfieri R. Salih A. M. Percario Z. A. Di Stefano M. (2025). Isolation and characterization of extracellular vesicles from EGFR mutated lung cancer cells. Clin. Exp. Med. 25 (1), 114. 10.1007/s10238-025-01643-w 40210802 PMC11985682

[B91] Schultz D. F. Billadeau D. D. Jois S. D. (2023). EGFR trafficking: effect of dimerization, dynamics, and mutation. Front. Oncol. 13, 1258371. 10.3389/fonc.2023.1258371 37752992 PMC10518470

[B92] Silva I. T. Carvalho A. Lang K. L. Dudek S. E. Masemann D. Durán F. J. (2015). *In vitro* and *in vivo* antitumor activity of a novel semisynthetic derivative of cucurbitacin B. PloS One 10 (2), e0117794. 10.1371/journal.pone.0117794 25674792 PMC4326133

[B93] Smeu A. Marcovici I. Dehelean C. A. Dumitrel S. I. Borza C. Lighezan R. (2025). Flavonoids and flavonoid-based nanopharmaceuticals as promising therapeutic strategies for colorectal cancer-an updated literature review. Pharmaceuticals 18 (2), 231. 10.3390/ph18020231 40006045 PMC11858883

[B94] Song Z. Shi Y. Han Q. Dai G. (2018). Endothelial growth factor receptor-targeted and reactive oxygen species-responsive lung cancer therapy by docetaxel and resveratrol encapsulated lipid-polymer hybrid nanoparticles. Biomed. and Pharmacother. = Biomedecine and Pharmacother. 105, 18–26. 10.1016/j.biopha.2018.05.095 29843041

[B95] Soonthonsrima T. Putra I. D. Phookphan P. Ei Z. Z. Yokoya M. Chanvorachote P. (2025). A promising resveratrol analogue suppresses CSCs in non-small-cell lung cancer via inhibition of the ErbB2 signaling pathway. Chem. Res. Toxicol. 38 (3), 415–432. 10.1021/acs.chemrestox.4c00436 40000408 PMC11921031

[B96] Soria J. C. Ohe Y. Vansteenkiste J. Reungwetwattana T. Chewaskulyong B. Lee K. H. (2018). Osimertinib in untreated EGFR-mutated advanced non-small-cell lung cancer. N. Engl. J. Med. 378 (2), 113–125. 10.1056/NEJMoa1713137 29151359

[B97] Sorkin A. Duex J. E. (2010). Quantitative analysis of endocytosis and turnover of epidermal growth factor (EGF) and EGF receptor. Curr. Protocols Cell Biology/Editorial Board, Juan S. Bonifacino 15, 14. 10.1002/0471143030.cb1514s46 20235100 PMC2878126

[B98] Sun X. L. Xiang Z. M. Xie Y. R. Zhang N. Wang L. X. Wu Y. L. (2022). Dimeric-(-)-epigallocatechin-3-gallate inhibits the proliferation of lung cancer cells by inhibiting the EGFR signaling pathway. Chemico-Biological Interact. 365, 110084. 10.1016/j.cbi.2022.110084 35970427

[B99] Tang J. C. Ren Y. G. Zhao J. Long F. Chen J. Y. Jiang Z. (2018). Shikonin enhances sensitization of gefitinib against wild-type EGFR non-small cell lung cancer via inhibition PKM2/stat3/cyclinD1 signal pathway. Life Sci. 204, 71–77. 10.1016/j.lfs.2018.05.012 29738778

[B100] Terai H. Hamamoto J. Emoto K. Masuda T. Manabe T. Kuronuma S. (2021). SHOC2 is a critical modulator of sensitivity to EGFR-TKIs in non-small cell lung cancer cells. Mol. Cancer Research MCR 19 (2), 317–328. 10.1158/1541-7786.MCR-20-0664 33106373

[B101] Tian X. Wang R. Gu T. Ma F. Laster K. V. Li X. (2022). Costunolide is a dual inhibitor of MEK1 and AKT1/2 that overcomes osimertinib resistance in lung cancer. Mol. Cancer 21 (1), 193. 10.1186/s12943-022-01662-1 36203195 PMC9535870

[B102] Torres-Martinez Z. Pérez D. Torres G. Estrada S. Correa C. Mederos N. (2023). A synergistic pH-Responsive serum albumin-based drug delivery system loaded with doxorubicin and pentacyclic triterpene betulinic acid for potential treatment of NSCLC. Biotech. Basel Switz. 12 (1), 13. 10.3390/biotech12010013 36810440 PMC9944877

[B103] Wada K. Lee J. Y. Hung H. Y. Shi Q. Lin L. Zhao Y. (2015). Novel curcumin analogs to overcome EGFR-TKI lung adenocarcinoma drug resistance and reduce EGFR-TKI-induced GI adverse effects. Bioorg. and Med. Chem. 23 (7), 1507–1514. 10.1016/j.bmc.2015.02.003 25753330 PMC4782611

[B104] Wang X. (2025). The effects of silibinin combined with EGFR-TKIs in the treatment of NSCLC. Cancer Med. 14 (3), e70643. 10.1002/cam4.70643 39907159 PMC11795421

[B105] Wang D. Bao B. (2020). Gallic acid impedes non-small cell lung cancer progression via suppression of EGFR-dependent CARM1-PELP1 complex. Drug Des. Dev. Ther. 14, 1583–1592. 10.2147/DDDT.S228123 32425504 PMC7186892

[B106] Wang J. Yang S. Cai X. Dong J. Chen Z. Wang R. (2016). Berberine inhibits EGFR signaling and enhances the antitumor effects of EGFR inhibitors in gastric cancer. Oncotarget 7 (46), 76076–76086. 10.18632/oncotarget.12589 27738318 PMC5342797

[B107] Wang Y. Liu Q. Chen H. You J. Peng B. Cao F. (2018). Celastrol improves the therapeutic efficacy of EGFR-TKIs for non-small-cell lung cancer by overcoming EGFR T790M drug resistance. Anti-Cancer Drugs 29 (8), 748–755. 10.1097/CAD.0000000000000647 29927769

[B108] Wang J. Sun P. Wang Q. Zhang P. Wang Y. Zi C. (2019). (-)-Epigallocatechin-3-gallate derivatives combined with cisplatin exhibit synergistic inhibitory effects on non-small-cell lung cancer cells. Cancer Cell Int. 19, 266. 10.1186/s12935-019-0981-0 31636509 PMC6791019

[B109] Wang X. J. Zhou R. J. Zhang N. Jing Z. (2019). 20(S)-ginsenoside Rg3 sensitizes human non-small cell lung cancer cells to icotinib through inhibition of autophagy. Eur. J. Pharmacol. 850, 141–149. 10.1016/j.ejphar.2019.02.023 30772396

[B110] Wang L. Zhang G. Qin L. Ye H. Wang Y. Long B. (2020). Anti-EGFR binding nanobody delivery system to improve the diagnosis and treatment of solid tumours. Recent Pat. Anti-Cancer Drug Discov. 15 (3), 200–211. 10.2174/1574892815666200904111728 32885759

[B111] Wang J. Xu C. Chen Y. Shao L. Li T. Fan X. (2021). β-elemene enhances the antitumor activity of erlotinib by inducing apoptosis through AMPK and MAPK pathways in TKI-resistant H1975 lung cancer cells. J. Cancer 12 (8), 2285–2294. 10.7150/jca.53382 33758606 PMC7974887

[B112] Wang T. H. Leu Y. L. Chen C. C. Li H. J. Yang S. C. Huang K. Y. (2022). Psorachromene induces apoptosis and suppresses tumor growth in NSCLC cells harboring EGFR L858R/T790M/C797S. Phytotherapy Research PTR 36 (5), 2116–2126. 10.1002/ptr.7432 35229911

[B113] Wang H. Du X. Liu W. Zhang C. Li Y. Hou J. (2024). Combination of betulinic acid and EGFR-TKIs exerts synergistic anti-tumor effects against wild-type EGFR NSCLC by inducing autophagy-related cell death via EGFR signaling pathway. Respir. Res. 25 (1), 215. 10.1186/s12931-024-02844-9 38764025 PMC11103851

[B114] Wang W. Kong M. Shen F. Li P. Chen C. Li Y. (2024). Ginsenoside Rg3 targets glycosylation of PD-L1 to enhance anti-tumor immunity in non-small cell lung cancer. Front. Immunol. 15, 1434078. 10.3389/fimmu.2024.1434078 39247194 PMC11377313

[B115] Wang J. Xian J. Zhang R. Wang Z. Zhang S. Zhao D. (2025). α-Mangostin exhibits antitumor activity against NCI-H1975 cells via the EGFR/STAT3 pathway: an experimental and molecular simulation study. Mol. Basel, Switz. 30 (6), 1294. 10.3390/molecules30061294 40142069 PMC11945449

[B116] Wang S. Lai Y. Huang H. Yuan J. Li S. Hui M. (2025). Exploring the antitumor effect of curcumin-piperlongumine hybrid molecule (CP) on EGFR-TKI-resistant non-small cell lung cancer using network pharmacological analysis and experimental verification. Acta Biochimica Biophysica Sinica 57, 1803–1813. 10.3724/abbs.2025076 40635549 PMC12666667

[B117] Wang Z. Xu W. Lei S. Lai Y. Zhang Y. Wang Y. (2025). A computer-aided, carrier-free drug delivery system with enhanced cytotoxicity and biocompatibility: a universal model for multifunctional lung cancer therapy. Colloids Surfaces. B, Biointerfaces 250, 114557. 10.1016/j.colsurfb.2025.114557 39933391

[B118] Wee P. Wang Z. (2017). Epidermal growth factor receptor cell proliferation signaling pathways. Cancers 9 (5), 52. 10.3390/cancers9050052 28513565 PMC5447962

[B119] Xia L. Wang J. Xue H. Li H. Li Q. Qin S. (2025). A CMTM6 nanobody overcomes EGFR-TKI resistance in non-small cell lung cancer. Adv. Sci. 12 (27), e2410945. 10.1002/advs.202410945 40521789 PMC12279249

[B120] Xiao X. He Z. Cao W. Cai F. Zhang L. Huang Q. (2016). Oridonin inhibits gefitinib-resistant lung cancer cells by suppressing EGFR/ERK/MMP-12 and CIP2A/Akt signaling pathways. Int. J. Oncol. 48 (6), 2608–2618. 10.3892/ijo.2016.3488 27082429

[B121] Xiao T. Zhu Y. Zhang L. Xiao K. Jia X. Liu Y. (2024). Griffithazanone A, a sensitizer of EGFR-targeted drug in Goniothalamus yunnanensis for non-small cell lung cancer. Heliyon 10 (19), e38489. 10.1016/j.heliyon.2024.e38489 39403494 PMC11471607

[B122] Xie C. Kong J. Miao F. Wang X. Sheng J. (2020). Combination effects of ellagic acid with erlotinib in a Ba/F3 cell line expressing EGFR H773_V774 insH mutation. Thorac. Cancer 11 (8), 2101–2111. 10.1111/1759-7714.13487 32525282 PMC7396384

[B123] Xie X. Zhan C. Wang J. Zeng F. Wu S. (2020). An activatable nano-prodrug for treating tyrosine-kinase-inhibitor-resistant non-small cell lung cancer and for optoacoustic and fluorescent imaging. Small Weinheim Der Bergstrasse, Ger. 16 (38), e2003451. 10.1002/smll.202003451 32815304

[B124] Xu S. W. Law B. Y. K. Mok S. W. F. Leung E. L. H. Fan X. X. Coghi P. S. (2016). Autophagic degradation of epidermal growth factor receptor in gefitinib-resistant lung cancer by celastrol. Int. J. Oncol. 49 (4), 1576–1588. 10.3892/ijo.2016.3644 27498688

[B125] Xu C. Jiang Z. B. Shao L. Zhao Z. M. Fan X. X. Sui X. (2023). β-Elemene enhances erlotinib sensitivity through induction of ferroptosis by upregulating lncRNA H19 in EGFR-mutant non-small cell lung cancer. Pharmacol. Res. 191, 106739. 10.1016/j.phrs.2023.106739 36948327

[B126] Yan X. Li P. Zhan Y. Qi M. Liu J. An Z. (2018). Dihydroartemisinin suppresses STAT3 signaling and Mcl-1 and survivin expression to potentiate ABT-263-induced apoptosis in non-small cell lung cancer cells harboring EGFR or RAS mutation. Biochem. Pharmacol. 150, 72–85. 10.1016/j.bcp.2018.01.031 29360439

[B127] Yang K. Chen Y. Zhou J. Ma L. Shan Y. Cheng X. (2019). Ursolic acid promotes apoptosis and mediates transcriptional suppression of CT45A2 gene expression in non-small-cell lung carcinoma harbouring EGFR T790M mutations. Br. J. Pharmacol. 176 (24), 4609–4624. 10.1111/bph.14793 31322286 PMC6965687

[B128] Ye M. X. Li Y. Yin H. Zhang J. (2012). Curcumin: updated molecular mechanisms and intervention targets in human lung cancer. Int. J. Mol. Sci. 13 (3), 3959–3978. 10.3390/ijms13033959 22489192 PMC3317752

[B129] Ye B. Chen P. Lin C. Zhang C. Li L. (2023). Study on the material basis and action mechanisms of Sophora davidii (Franch.) skeels flower extract in the treatment of non-small cell lung cancer. J. Ethnopharmacol. 317, 116815. 10.1016/j.jep.2023.116815 37400006

[B130] Yi K. Zhou Y. Zhang M. Guo Y. (2022). The core mechanism of yiqi yangjing decoction inhibiting nonsmall-cell lung cancer. evidence-based complementary and alternative medicine. eCAM 2022, 2256671. 10.1155/2022/2256671 35586682 PMC9110163

[B131] Zaryouh H. Van Loenhout J. Peeters M. Vermorken J. B. Lardon F. Wouters A. (2022). Co-Targeting the EGFR and PI3K/Akt pathway to overcome therapeutic resistance in head and neck squamous cell carcinoma: what about autophagy? Cancers 14 (24), 6128. 10.3390/cancers14246128 36551613 PMC9776372

[B132] Zhang L. Tao X. Fu Q. Ge C. Li R. Li Z. (2019). Curcumin inhibits cell proliferation and migration in NSCLC through a synergistic effect on the TLR4/MyD88 and EGFR pathways. Oncol. Rep. 42 (5), 1843–1855. 10.3892/or.2019.7278 31432177 PMC6775800

[B133] Zhang D. Zhang T. Zhang Y. Li Z. Li H. Zhang Y. (2022). Screening the components of Saussurea involucrata for novel targets for the treatment of NSCLC using network pharmacology. BMC Complementary Medicine Therapies 22 (1), 53. 10.1186/s12906-021-03501-0 35227278 PMC8886885

[B134] Zhang J. Li C. Li W. Shi Z. Liu Z. Zhou J. (2024). Mechanism of luteolin against non-small-cell lung cancer: a study based on network pharmacology, molecular docking, molecular dynamics simulation, and *in vitro* experiments. Front. Oncol. 14, 1471109. 10.3389/fonc.2024.1471109 39582546 PMC11582065

[B136] Zhang R. Zheng Y. Zhu Q. Gu X. Xiang B. Gu X. (2024). β-Elemene reverses gefitinib resistance in NSCLC cells by inhibiting lncRNA H19-Mediated autophagy. Pharm. Basel, Switz. 17 (5), 626. 10.3390/ph17050626 38794196 PMC11124058

[B137] Zhang Q. Li N. Ma X. Qiu Y. Chen Y. (2025). Berberine protects against gefitinib-induced liver injury by inhibiting the HMGB1/TLR4/NF-κB pathway. Front. Pharmacol. 16, 1645634. 10.3389/fphar.2025.1645634 40932871 PMC12417729

[B138] Zhao X. P. Zheng X. L. Huang M. Xie Y. J. Nie X. W. Nasim A. A. (2023). DMU-212 against EGFR-mutant non-small cell lung cancer via AMPK/PI3K/Erk signaling pathway. Heliyon 9 (5), e15812. 10.1016/j.heliyon.2023.e15812 37305501 PMC10256861

[B139] Zhao L. P. Wang H. J. Hu D. Hu J. H. Guan Z. R. Yu L. H. (2024). β-Elemene induced ferroptosis via TFEB-mediated GPX4 degradation in EGFR wide-type non-small cell lung cancer. J. Adv. Res. 62, 257–272. 10.1016/j.jare.2023.08.018 37689240 PMC11331178

[B140] Zhou Y. Huang S. Guo Y. Ran M. Shan W. Chen W. H. (2023). Epigallocatechin gallate circumvents drug-induced resistance in non-small-cell lung cancer by modulating glucose metabolism and AMPK/AKT/MAPK axis. Phytotherapy Research PTR 37 (12), 5837–5853. 10.1002/ptr.7990 37621136

[B141] Zhou R. Liu Z. Wu T. Pan X. Li T. Miao K. (2024). Machine learning-aided discovery of T790M-mutant EGFR inhibitor CDDO-Me effectively suppresses non-small cell lung cancer growth. Cell Communication Signaling CCS 22 (1), 585. 10.1186/s12964-024-01954-7 39639305 PMC11619116

[B142] Zhou F. Guo H. Xia Y. Le X. Tan D. S. W. Ramalingam S. S. (2025). The changing treatment landscape of EGFR-mutant non-small-cell lung cancer. Nat. Rev. Clin. Oncol. 22 (2), 95–116. 10.1038/s41571-024-00971-2 39614090

[B143] Zhu Y. He W. Gao X. Li B. Mei C. Xu R. (2015). Resveratrol overcomes gefitinib resistance by increasing the intracellular gefitinib concentration and triggering apoptosis, autophagy and senescence in PC9/G NSCLC cells. Sci. Rep. 5, 17730. 10.1038/srep17730 26635117 PMC4669414

[B144] Zi C. T. Wu Y. L. Liu Z. H. Niu Y. Yuan W. J. Yang Z. W. (2025). Novel (-)-eigallocatechin-3-gallate-erlotinib conjugates via triazole rings inhibit non-small cell lung cancer cells through EGFR signaling pathway. Bioorg. Chem. 157, 108263. 10.1016/j.bioorg.2025.108263 39938444

